# Engineered bacteria: Strategies and applications in cancer immunotherapy

**DOI:** 10.1016/j.fmre.2024.11.001

**Published:** 2024-11-13

**Authors:** Shuhao Zhang, Rui Li, Yunxue Xu, Renfa Liu, Desheng Sun, Zhifei Dai

**Affiliations:** a Department of Biomedical Engineering, College of Future Technology, National Biomedical Imaging Center, Peking University, Beijing 100871, China; b Department of Ultrasonic Imaging, Peking University Shenzhen Hospital, Shenzhen 518035, China

**Keywords:** Drug delivery, Tumor targeting, Synthetic biology, Engineered bacteria, Genetic circuits, Immunotherapy

## Abstract

Cancer therapy remains a critical medical challenge. Immunotherapy is an emerging approach to regulating the immune system to fight cancer and has shown therapeutic potential. Due to their immunogenicity, bacteria have been developed as drug-delivery vehicles in cancer immunotherapy. However, ensuring the safety and efficacy of this approach poses a considerable challenge. This paper comprehensively explains the fundamental processes and synthesis principles involved in immunotherapy utilizing engineered bacteria. Initially, we list common engineered strains and discuss that growth control through genetic mutation promises therapeutic safety. By considering the characteristics of the tumor microenvironment and the interaction of specific molecules, the precision targeting of tumors can be improved. Furthermore, we present a foundational paradigm for genetic circuit construction to achieve controlled gene activation and logical expression, directly determining drug synthesis and release. Finally, we review the immunogenicity, the expression of immunomodulatory factors, the delivery of immune checkpoint inhibitors, and the utilization of bacteria as tumor vaccines to stimulate the immune system and facilitate the efficacy of cancer immunotherapy.

## Graphical abstract

**Figure:**
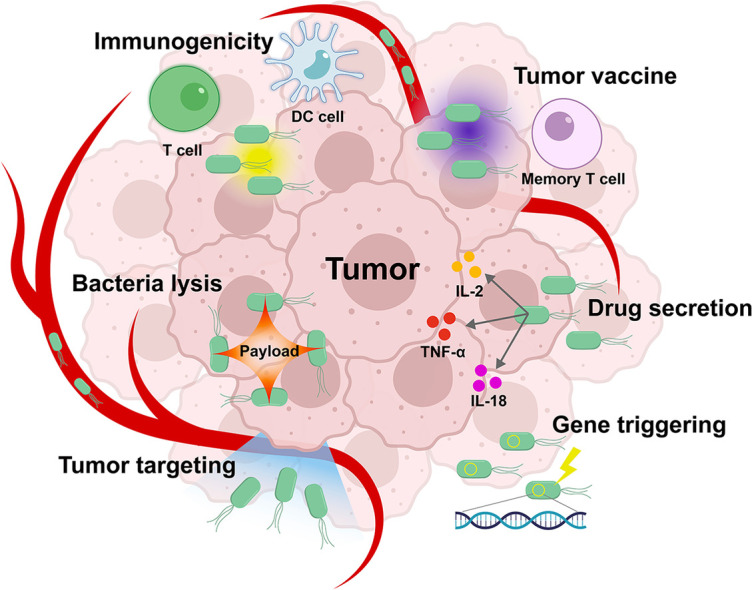

## 1 Introduction

Cancer is a spectrum of diseases characterized by the uncontrolled proliferation and spread of abnormal cells throughout the body. These cells form tumors, infiltrate neighboring tissues and spread to other anatomical sites via the bloodstream or lymphatic system. Cancer symptoms vary depending on the type and stage of the disease, including pain, fatigue, weight loss, changes in the skin, and changes in bowel or bladder habits. The cancer diagnostic methods typically include imaging techniques (e.g., X-ray imaging, computed tomography, and magnetic resonance imaging), hematologic analyses, and histopathological scrutiny through biopsy procedures [1]. For cancer therapy, the interventions span a range of modalities, comprising surgical excision, radiotherapy [2], chemotherapy [3], targeted therapy [4], and immunotherapy [5]. Compared with traditional treatments, immunotherapy has the advantages of high specificity and low side effects. This method is based on the immune response principles, utilizing biologically active molecules and employing preventive, supportive, and regulatory measures to activate and enhance the anti-cancer function. The conception of cancer immunotherapy may trace its origins to the interplay between disease infection and tumor regression. Particularly noteworthy is the phenomenon wherein pathogenic microorganisms and viral infections induce the suppression or even complete disappearance of tumor growth, prompting the attention and scrutiny of immunologists. As early as the 19th century, William B. Coley performed the first cancer immunotherapy. He primarily treated patients with inoperable bone and soft-tissue sarcomas using bacterial toxins, noting that this method was significantly less effective on other cancer types [6]. However, constrained by the nascent developments in immunology, the mechanism of action of Coley's toxins remained unclear. Coupled with the heightened risk of infection posed by highly pathogenic bacteria, Coley's toxins are not widely applied [7]. This therapy was controversial at that time, but it was an attempt to use bacteria for cancer immunotherapy. Moreover, modern immunological studies have shown that bacteria activate the immune system and thereby cause tumor regression. His notable contributions have earned him the epithet ‘Father of Immunotherapy’ [8].

Compared to traditional drug delivery methods, bacteria as therapeutic delivery vehicles offer several distinct advantages. First, as living organisms, bacteria possess the ability to specifically target tissues or microenvironments, penetrating deeply into hypoxic or necrotic regions of tumors that are often inaccessible to conventional therapies [9]. This targeting capability enhances treatment precision. Second, bacteria are highly amenable to genetic modification, allowing them to express a diverse range of therapeutic agents, including small molecules, proteins, and nucleic acids, making them a versatile platform for drug delivery [10]. Additionally, bacteria can provide sustained drug release, maintaining a high local concentration of therapeutic agents at the disease site, thereby minimizing systemic toxicity and potentially reducing dosing frequency [11]. Finally, bacteria are natural immune activators, and in the context of cancer immunotherapy, they can stimulate the host's immune system, further enhancing the efficacy of the delivered therapeutics. These characteristics make bacteria-based delivery systems a promising alternative for the treatment of complex diseases, such as cancer, where precise delivery and immune modulation are critical for therapeutic success [12]. Bacteria activate an innate immune response against the pathogen, involving neutrophils, natural killer cells, macrophages, and dendritic cells, strengthening the response to T lymphocytes and thereby triggering adaptive immunity. Therefore, the immunogenicity makes the bacteria suitable for immunotherapy. Bacillus Calmette-Guérin (BCG) therapy, using a live attenuated strain of *Mycobacterium bovis*, is one of the most successful examples of cancer immunotherapy. It is the gold-standard treatment for non-muscle-invasive bladder cancer (NMIBC), inducing a durable and effective antitumor immune response [13]. Hence, the concept of engineering bacteria to precisely target tumors, proliferate within them, and elicit an immune response for cancer treatment appears to be a viable proposition. Nevertheless, it is crucial to acknowledge that natural bacteria are typically pathogenic and inherently lack tumor-specific therapeutic effects. Additionally, the implementation of this technology faces significant challenges, including safety considerations, precise targeting, controllability, and therapeutic effectiveness, which warrant comprehensive exploration and resolution.

Engineering bacteria using synthetic biology or advanced materials approaches can help address these issues. Advancements in synthetic biology render the concept of engineered bacteria for tumor immunotherapy both plausible and promising [14]. Employing approaches rooted in engineering principles, synthetic biologists undertake artificial modifications of biological systems. This methodology is designed to enhance native biosystems and create novel signaling pathways within intricate biological networks. Their research is based on the genetic central dogma to effect artificial interventions across various gene expression levels. The operational paradigm can be abstracted into three essential steps: signal reception, processing and computation, and output for responsive actions [15]. It also achieves the modularization and standardization of internal cellular components, facilitating the design of fundamental units for artificial biological systems [16,17]. Unlike the inside-out approach of synthetic biology, material modification involves the application of advanced materials to confer diverse physical and chemical properties to the bacterial surface. The integration with modern treatment technologies serves to surmount the inherent limitations of bacterial therapy, expanding the function of bacterial vectors [18]. These technologies help live bacteria achieve diverse and effective cancer immunotherapy under the premise of safety. Synthetic circuits enable the release of therapeutic payloads, facilitating diverse immunotherapeutic strategies, including the expression of effector protein, delivery of therapeutic drugs, and production of tumor vaccines [12]. The orchestrated approach epitomizes bacterial-based tumor immunotherapy. In this review, aimed at the safety and efficacy of treatment, we review the basic principles and methods of engineered bacteria from the aspects of safety, targeting, logic circuit, and treatment strategy and summarize the basic paradigm of this therapy. Finally, based on the evidence of host-bacteria interaction, we propose new insights into the utilization of engineered live bacteria for cancer immunotherapy.

## 2 Engineering methods for safety

Engineered bacteria with diverse traits for cancer therapy demonstrate native anticancer effects through direct cell killing and immune system activation. Overcoming safety challenges necessitates essential virulence reduction in these bacteria as a prerequisite for effective cancer immunotherapy. It is vital to design immunotherapy strategies according to the characteristics of the strain, reduce side effects through genetic modification, and improve the safety of treatment by controlling its growth and metabolism.

### 2.1 Diverse strains and bacterial native anticancer effects

With advances in synthetic biology and molecular biology, various strains have been engineered for tumor immunotherapy. *Bifidobacterium infanti*, a gram-positive anaerobic bacterium derived from human infant feces, is non-pathogenic and exhibits minimal side effects upon administration to animals, irrespective of the route of administration [19]. *Salmonella* spp. can disseminate to systemic organs, taking residence in local macrophages, thereby inducing both humoral and cellular immune responses. Due to its effective tumor-targeting capabilities, engineered *Salmonella* spp. has been extensively investigated [20,21]. *Clostridium* spp. produce endospores that resist harsh environmental conditions, such as high temperatures, dehydration, low-energy radiation, and disinfectants. These spores selectively germinate within the hypoxic and necrotic regions of solid tumors, showing remarkable specificity [22]. *Listeria* spp., which localizes in the cytosol, has been regarded as an appealing vector for *in vivo* delivery of tumor-associated antigens (TAA) to fight cancer for a long time [23]. Additional strains employed in cancer immunotherapy include *Caulobacter* [24], *Proteus* [25], and *Streptococcus* [26] among others. The selection of strains should align with the chosen immunotherapy strategy and their potential to produce a therapeutic effect effectively. Emphasis should be placed on the use of safe, non-pathogenic microorganisms whenever possible. For instance, *Escherichia coli* Nissle 1917 (EcN), a gram-negative probiotic isolated initially during World War I [27], is serum-sensitive and lacks the production of enterotoxins or cytotoxins associated with pathogenic *E. coli* strains [28]. Numerous studies indicate that EcN-mediated tumor therapies successfully regress tumors and enhance survival in mice, which enables EcN to be a suitable probiotic for diverse clinical applications as a living therapeutics [29].

Nevertheless, the virulence of bacteria is usually unavoidable. The virulence depends on their invasiveness into the host and the presence of bacterial toxins. Bacteria gain entry through various routes, attaching to the digestive tract, respiratory tract, or other mucous membranes and epithelium, facilitated by fimbriae. The capacity to proliferate and evade the immune system is crucial for successful invasion and infection. To summarize the virulence factors of bacteria, Chen et al. established the relevant database VFDB [30]. The adverse effects of bacteria allow them to kill tumor cells directly. It has been reported that natural bacteria confer benefits in terms of tumor regression and extended survival time. *Listeria* activates NADPH oxidase to promote the production of ROS, which induces the death of breast cancer cells 4T1 and MCF7 [31]. In addition, the bacteria further activate the immune system by their immunogenicity, providing a native anti-cancer effect. In mice bearing orthotopic hepatoma, the number of *S. choleraesuis* increased with tumor growth. The accumulation and amplification of bacteria significantly repressed tumor growth and prolonged the survival time [32]. The infection of *S.pyogenes* caused a quantitative reduction in tumor size and led to frequent ulceration and apparent necrosis, resulting in tumor regression [26]. Bacterial infections can be harmful or even fatal, posing a significant challenge to therapeutic safety. Consequently, engineering bacteria to diminish virulence becomes an essential prerequisite.

### 2.2 Genetic mutation is the basic principle of toxicity reduction

Enhancing safety primarily involves genetic modification and bacterial distribution control. The strategy for diminishing bacterial virulence revolves around disrupting genes essential for bacterial survival and specific pathogenic functions. Initially, genetic modification aims to decrease toxin expression, decreasing side effects during treatment. Subsequently, optimizing the preferential growth of living organisms in tumor areas necessitates the knockout of genes linked to bacterial nutrition and the chemical components of the tumor microenvironment (TME) in normal tissues. This renders bacterial growth and reproduction reliant on external nutritional sources, effectively limiting adverse effects (Table 1).

**Table 1:** **Representative genetic modification methods for *Salmonella* and *Listeria* attenuated strains**.

Strain name	Genetic modification	Phenotype description	Refs
*Salmonella* spp.			

VNP20009	ΔpurI/ΔmsbB	Lipid A structural modification; Reduced ability to induce TNF-α; Lack of adenine synthesis	[33,34]
ΔppGpp	ΔrelA/ΔspoT	Inability to produce ppGpp (global regulator of bacterial adaptation to extreme environments); reduced bacterial invasion	[35,36]
A1-R	Δleu/Δarg	Auxotrophic strain defective in leucine and arginine synthesis	[37,38]
SL3261	ΔaroA	Defective in aromatic amino-acid biosynthesis	[46]
LH430	ΔphoP/ΔphoQ	Decreases survival in macrophages and increases sensitivity to antimicrobial peptides and reduces ability to modify Lipid A	[47]
YB1	Δasd	Defective in diaminopimelic acid (DAP) synthesis, leading to bacterial lysis during growth without an exogenous DAP supply	
C4550	Δcya/Δcrp	Disabled production of cAMP (cyclic adenosine monophosphate) synthetase and cAMP receptor protein	[48]

*Listeria* spp.			

DP-L4027	ΔLLO (hly)	Defective phagolysosome release	[49]
DP-L4029	ΔactA	Defective surface-bound ActA polypeptide, constitutive LLO activity at physiologic pH	[50]
DP-L4017	LLO L461T, LLOD26	Cytotoxic, defective cell-to-cell spreading	[51]
DP-L4042	ΔPEST	Cytotoxic, defective cell-to-cell spreading	[52]
DP-L4364	ΔlplA	Unable to produce lipoate protein ligase, limited ability to proliferate intracellularly	[53]
CS-L0001	ΔactA/ΔinlB	No host actin nucleation, defective cell-to-cell spreading	[41,42]

For *Salmonella* spp., the endotoxin lipid A can cause septic shock by producing large amounts of tumor necrosis factor α (TNF-α). It has been shown that the modified bacteria VNP20009 not only has antitumor effects but also that mutations in the *msbB* gene can alter lipid A and thus significantly reduce the induction of TNF-α without affecting the targeting of the bacteria to the tumor and its accumulation in the tumor. Mutation in the *purI* gene of VNP20009 also affects adenine synthesis, which is an artificially designed nutritional defect [33,34]. Another attenuated *Salmonella* strain, ∆ppGpp, is engineered by regulating endotoxin gene expression. This strain, a double mutant (*relA^−^, spoT^−^*) with defects in ppGpp synthesis, results in downregulated endotoxin gene expression. Consequently, it exhibits reduced virulence in mice following systemic infection and protects them from challenges with wild-type *Salmonella* [35,36]. A1-R, an auxotrophic strain, is defective in synthesizing leucine and arginine, which have double mutants (*leu^−^, arg^−^*). These nutrients are high in the tumor and, therefore, allow the enrichment of the A1-R strain within the tumor [37,38]. Similarly, YB1 and ST8 strains are defective in diaminopimelic acid (DAP) synthesis because of *asd^−^* mutation [39,40]. For *Listeria* spp., the measures to reduce virulence are diverse and delicate. The suppression of cell-to-cell spreading improves its treatment safety. Internalin B mediates direct infection by non-phagocytic cells, and ActA mediates indirect infection by intercellular transmission from adjacent phagocytic cells. By deleting both ActA (*actA^−^*) and Internalin B (*inlB^−^*), the immune potency of *Listeria* is maintained, and its virulence is attenuated *in vivo* [41,42]. Listeriolysin O (LLO), a cholesterol-dependent cytolysin, facilitates its escape from phagosomes, allowing the bacteria to proliferate within the host [43]. Truncated immunogenic LLO in *Lm*-LLO strain can be fused with the antigens, such as prostate-specific antigen (PSA) and HPV-16 E7 protein(E7), making these strains into tumor vaccines and non-vaccine immunotherapy carriers [44,45]. The principles and methods employed to reduce virulence are applicable to various bacteria, allowing for cross-referencing the knockout of essential growth-related genes across different strains. It is crucial to underscore that the modification of bacterial virulence needs to be seamlessly integrated with tumor targeting and immunotherapy strategies. Additionally, approaches addressing nutritional deficiencies and attenuating measures should be tailored based on the specific cancer types under consideration.

### 2.3 Imaging tools for detecting engineered bacteria

Genetic modification is the modification of the physiological properties of the bacteria, and to ensure safety, the distribution of engineered bacteria *in vivo* must also be detected and traced with the help of imaging technology. Currently, the main imaging techniques are fluorescence, bioluminescence, magnetic resonance imaging (MRI), positron emission tomography (PET) and ultrasound imaging [54]. By transfecting plasmids encoding fluorescent proteins into bacteria, they can be imaged and traced in the presence of excitation light [55]. Zheng et al. administered GFP-labelled *Salmonella* via intravenous injection to tumor-bearing mice. They found that GFP fluorescence from the bacteria was abundant in tumors, but significantly reduced in the liver [56]. Compared to fluorescent proteins, bioluminescent elements such as LuxCDABE [57], firefly luciferase, and other reporter gene systems are more suitable for *in vivo* imaging of small animals. One benefit of luminescence compared to GFP fluorescence is that, when a suitable substrate is present, the emitted light (480 nm) from luminescence can effectively penetrate the tissues of living organisms. In comparison, GFP requires external blue light for excitation, which can only penetrate tissues to a depth of about 2 mm. As a result, fluorescence cannot be observed in deeper tissues of intact animals [58]. These optical imaging techniques have excellent validity and sensitivity in animal models [59]. However, their clinical application is very limited due to the weak penetration of visible light in human tissues and the lack of optical imaging devices for humans. For in-depth imaging and monitoring, MRI and PET technologies with higher sensitivity and resolution are more suitable for clinical applications. Benoit et al. examined whether *Magnetospirillum magneticum* AMB-1 can target tumors in mice and provide positive MRI contrast. Engineered to produce 25 nm magnetite particles, AMB-1 was tested *in vitro* and *in vivo*. Tumor targeting was confirmed using ^64^Cu-labeled bacteria and PET imaging. Results showed that after injection, AMB-1 colonized tumors and was cleared from other organs by day 6. Increased tumor contrast suggested its potential for improved tumor imaging [60]. Mowday et al. demonstrated that the bacterial nitroreductase NfsA from *E. coli* (NfsA_Ec) can serve as an effective reporter gene for non-invasive PET imaging. NfsA_Ec metabolizes 2-nitroimidazole-based PET agents like EF5 and ^18^F-HX4, enabling visualization of gene expression *in vitro* and *in vivo* [61]. Ultrasound imaging is well suited for mass diffusion and clinical translation due to its convenience, low cost, safety and other advantages over MRI and PET. Gas vesicles (GVs) are unique, genetically encoded, hollow protein nanostructures that can be used by photosynthetic bacteria or archaea to achieve cell buoyancy. The GVs are composed of an amphiphilic protein shell, measuring 45–250 nm in width and 100–600 nm in length. Because of the presence of hydrophobic surfaces in the airbag, the airbag only allows gas penetration and excludes liquid water [62,63]. Recent studies have found that GVs can be used as contrast agents for techniques such as high-frequency diagnostic ultrasound and MRI, as well as for non-invasive imaging of gene expression [64]. Hurt et al. of Shapiro's team identified two acoustic reporter genes (ARGs) for bacterial and mammalian cells, respectively, as a result of a phylogenetic screen of candidate gas vesicle gene clusters from different bacteria and archaea, and improved them to enhance their non-linear contrast. Using these GVs, non-invasive imaging of bacterial gene expression for in situ tumor colonization and tumor homing therapy was achieved to track tumor gene expression and growth progression in breast cancer model mice [65].

## 3 Tumor targeting strategies

The targeting of bacteria to tumor tissue is a precondition for immunotherapy. The accurate targeting of tumor tissue by bacteria depends on both the tropism inherent in the specific tumor microenvironment and the artificial modification for bacterial characteristics. The molecular characteristics exhibited on the surface of cancer cells contribute to enhancing the precision and effectiveness of the adhesion. When coupled with ultrasound or magnetic fields, or through the migration of immune cells, the directional impetus for bacterial targeting is further intensified.

### 3.1 Microenvironment-mediated bacterial orientation

**Fig. 1:**
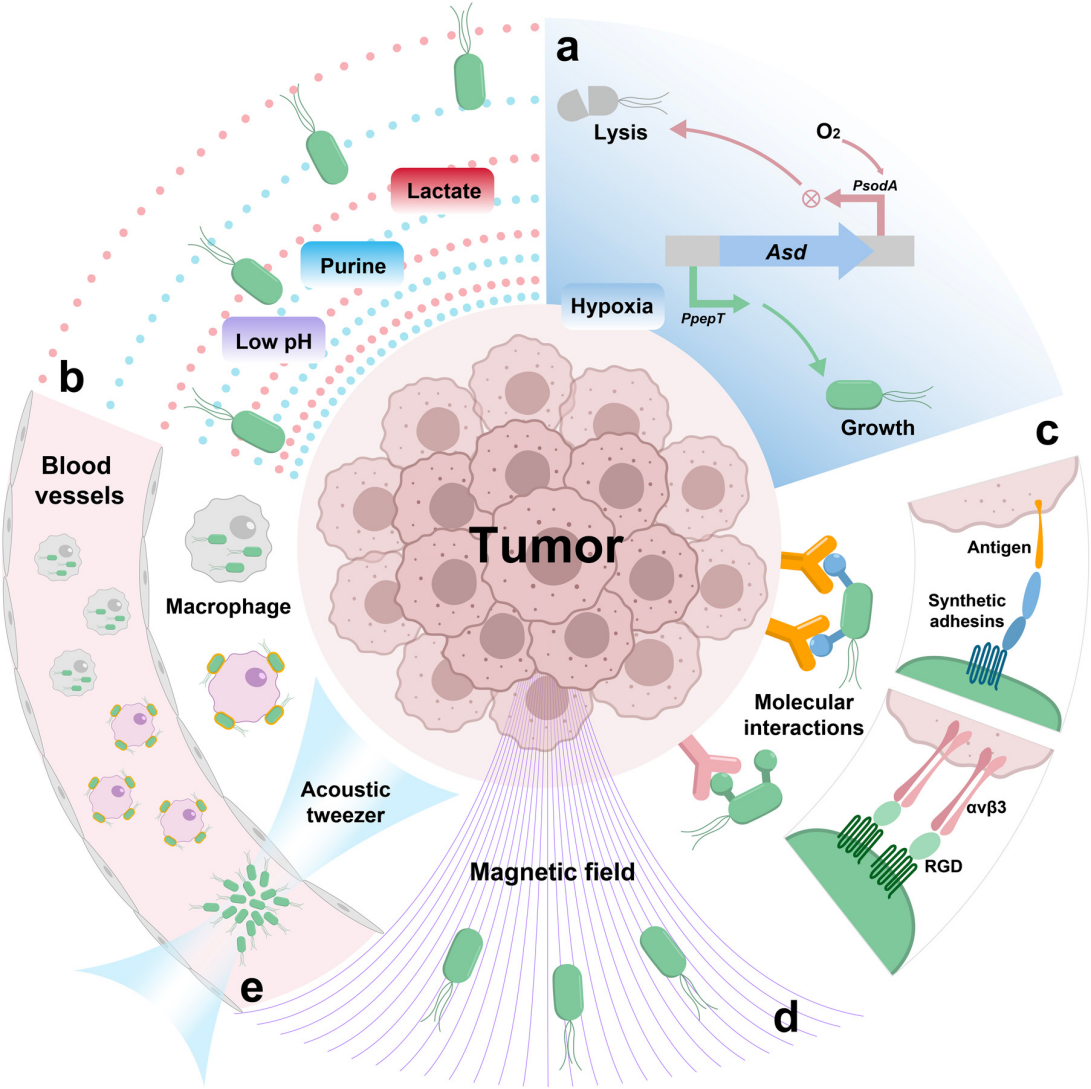
**Basic strategies for targeting tumors by engineered bacteria.** (a) Hypoxia induces asd gene expression and bacterial normal growth. Aerobic induction of asd antisense mRNA production causes bacterial lysis. pPepT, hypoxia-induced promoter. pSodA, aerobic-induced promoter. asd, one of the critical genes in bacterial peptidoglycan synthesis. (b) Chemicals such as acid, purine and lactate induce bacterial enrichment in tumors. (c) Bacterial molecular interactions with tumor cells promote specific targeting. RGD, a tripeptide sequence consisting of L-arginine, glycine and L-aspartic acid. α_v_β_3_, a type of integrin that is a receptor for RGD. (d) Use of magnetic fields to direct magnetotropic bacteria towards tumor enrichment. (e) Adjunctive methods such as macrophage transport and acoustic tweezers to control bacterial movement.

The tumor microenvironment serves as the nurturing substrate for tumors, encompassing diverse cell types, tumor blood vessels, secreted factors, and the extracellular matrix. The heightened metabolic demands of tumor cells result in unique characteristics such as low oxygen levels, increased acidity, and higher reactive oxygen species [66]. Unlike normal tissue, the supply of oxygen and other vital nutrients by blood vessels in tumors is uneven, thus leading to hypoxia and necrosis [67]. Hypoxia provides a conducive environment for anaerobic bacteria to target and colonize tumors selectively. To reinforce the effect of oxygen concentration, the hypoxic signal can be coupled to the survival of bacteria, making hypoxia a necessary condition for their growth. Since anaerobic bacteria can adapt to an anoxic environment, hypoxia can serve as a condition for anaerobic bacteria to target tumors and colonize. Yu et al. constructed the initial form of a hypoxia-responsive sensor based on the YB1 strain synthesized by *Salmonella typhimurium*. They connect the hypoxia-inducible promoter, pPepT, to the gene asd, which is essential for the survival of bacteria, and reverse the linkage to the aerobic-inducible promoter, pSodA. This design allows hypoxia to be the targeting signal, and bacteria undergo lysis under aerobic conditions and survive under anaerobic conditions. Oxygen conditions are directly coupled to bacterial survival, which provides the effect of a hypoxia response and improves safety. The YB1 strain has an innovative design that enhances safety, makes hypoxia a signal for targeting, and affects the eventual colonization of the bacteria (Fig. 1a) [39].

Chemical components within the tumor microenvironment also contribute to the targeting process. In the *in vitro* tumor model experiments, *Salmonella* can gravitate toward small molecule nutrients such as aspartic acid, serine and ribose. In animal models, dying cells accelerate the growth rate of *Salmonella* [68,69]. The nutritional deficiency of bacteria and tumor targeting are complementary; as previously mentioned, VNP20009 was further deleted with the *purI* gene, allowing them to colonize purine-rich tissues such as tumors [33]. Similarly, responses to lactate concentration [70,71] and pH [72,73] have also been developed as biosensors, which provide modular tools for precise tumor targeting and controlled gene expression (Fig. 1b). While a single factor can enable bacteria to attain tumor tropism, they may still survive and maintain a particular abundance in normal tissues. This means that the difference in bacterial abundance between tumors and normal tissue is insignificant, and neither therapeutic safety nor efficacy can be guaranteed. The essence of targeting is the control of bacterial growth. Ideally, bacteria should be substantially enriched in the tumor and distributed orders of magnitude lower in normal tissue than in the tumor. Achieving this requires the strategic combination of different environmental factors to curtail the non-specific invasion of normal tissues by bacteria significantly. To confine bacterial growth to specific tissues or regions in the body, Chien et al. demonstrate the construction of bacterial biosensors that can sense oxygen, pH, and lactate levels, and show that engineered bacteria with these biosensors exhibit preferential growth in physiologically relevant conditions. They also develop containment strains by coupling bacterial growth with the expression of essential genes controlled by the biosensor promoters, resulting in selective bacterial growth under specific environmental conditions. The authors further enhance the specificity of the biocontainment circuits by designing an AND logic gate circuit that requires the presence of two different environmental conditions for bacterial replication. The findings suggest that genetically programmed biosensors and containment strains have the potential to improve the localization of bacteria to specific niches in the body, which could have implications for therapeutic applications [74].

### 3.2 Molecular interactions enhance specific tumor targeting

To improve targeting efficacy, an effective strategy involves the interaction between ligands expressed by bacteria and receptors on the membrane surface of cancer cells, thereby enhancing the specificity and precise targeting of a specific class of tumor cells (Fig. 1c). As mentioned above, the capacity of bacteria to attach to the host affects their invasiveness and virulence as pathogens. Adhesins such as intimin and invasin are crucial virulence factors involved in the attachment and effacement processes of bacteria. *E. coli*, known for causing diarrhea, adheres to the colonic epithelium, leading to the formation of actin pedestals and disruption of microvilli on enterocytes [75]. Intimin, crucial for bacterial attachment, has been engineered by replacing certain domains (such as D1, D2, and D3) while retaining essential elements like the signal peptide (SP), lysin motif (LysM), β-barrel, and domains D00 and D0, which form its anchoring module [76]. Expression of synthetic adhesins directed the specific adhesion of bacteria to abiotic surfaces and mammalian cells expressing on their surface the target molecule recognized [77]. Piñero-Lambea et al. reported synthetic adhesins (SAs), which have a modular structure comprising a stable β-domain for outer membrane anchoring and surface-exposed immunoglobulin domains with high affinity. Depending on the tumor class, the VHH region of synthetic adhesins can be designed to target multiple antigens, which embody the modularization method [78]. Synthetic adhesion molecules expressed on the bacterial outer membrane can specifically recognize and interact with tumor antigens through an immunoglobulin domain, significantly reducing off-target colonization. Moreover, proteins containing the Arg-Gly-Asp (RGD) attachment site, along with integrins as their receptors, form a critical recognition system for cell adhesion. Since integrin-mediated attachment deeply affects cell migration, growth, differentiation, and apoptosis, RGD peptides are considered potential therapeutic agents for conditions like thrombosis, osteoporosis, and cancer [79]. Extending this principle, the interaction of RGD peptides with integrin can also be engineered for bacteria-to-cell recognition and attachment. *Salmonella* with outer membrane protein A (OmpA) integrated with RGD peptide sequence binds tightly to α_v_β_3_ integrin overexpressing cancer cells but binds weakly to α_v_β_3_-negative cancer cells. *In vivo* studies indicate that *Salmonella* featuring RGD exhibits robust targeting efficiency, fostering tumor regression and extending survival in murine models of breast cancer and melanoma [80].

### 3.3 Ancillary methods for precise delivery of bacteria

In order to make bacterial movement more direct and clear, certain auxiliary methods facilitate their direct transportation to the tumor site. Magnetotactic bacteria, displaying morphological, metabolic, and phylogenetic diversity, share a common characteristic—the ability to biomineralize membrane-encased, single-magnetic-domain mineral crystals known as magnetosomes. These magnetosomes induce cellular orientation along the Earth's geomagnetic field. Among magnetotactic bacteria, *Magnetotactic cocci* represents the most frequently observed type, forming a distinct phylogenetic group [81]. *Magnetococcus marinus* (MC-1) exhibits a unique behavior, moving along magnetic field lines toward hypoxic environments. Utilizing a magnetic field to guide MC-1 bacteria toward the hypoxic microenvironment of tumors enables effective tumor targeting. While MC-1 lacks the direct capability to kill tumor cells, researchers utilized its capacity to transport liposomes. Intriguingly, this modification minimally affected their magnetotactic ability. The exhibited magneto-aerotactic behavior suggests a promising approach to substantially improve the therapeutic index of diverse nanocarriers in tumor hypoxic regions [81,82]. Another strain, *Magnetospirillum magneticum* (AMB-1), may induce apoptosis in cancer cells through iron competition in the TME [83]. AMB-1 interferes with cancer cell proliferation by chelating iron, leading to increased apoptosis. Integrating this strain with existing chemotherapeutic drugs could significantly enhance current bacterial cancer therapy strategies. (Fig. 1d) [84]. Nevertheless, magnetic fields exhibit restricted focusing capabilities, and the use of ferromagnetic labeling may have adverse effects on bacterial survival. Leveraging the superior penetration and focusing of ultrasound, acoustic tweezers represent a versatile toolset for manipulating bioparticles, ranging from nanometer-sized extracellular vesicles to millimeter-sized multicellular organisms [85]. Yang and colleagues have developed a phased-array holographic acoustic tweezer manipulation technique. This technique achieves non-invasive, precise manipulation and efficient enrichment of bacteria clusters with gas vesicles in biological organisms and blood flow. In animal models, it has been applied to achieve targeted treatment for tumors (Fig. 1e) [86].

*Listeria monocytogenes* is a gram-positive facultative intracellular bacterium that uniquely resides in the cytoplasm of host cells. Intriguingly, it enters host cells through phagocytosis but escapes into the cytoplasm by disrupting the phagosomal membrane, primarily via the action of the secreted virulence factor listeriolysin O (LLO) [87]. This characteristic positions them as potential vectors and vaccine platforms for directing antigens to the major histocompatibility complex (MHC) class I pathway of antigen processing, generating authentic CTL epitopes [[88], [89]]. Myeloid-derived suppressor cells, comprising immature granulocytes, macrophages, and DC [90], can potentially serve as efficient bacterial carriers [91]. An intimate relationship exists between *Listeria* and myeloid-derived suppressor cells (MDSCs), as they migrate to the primary tumor, activating immune suppressive factors. In addition, MDSCs can be used as cellular missiles to deliver Listeria to neoplastic lesions selectively. *Listeria* within MDSCs remains unaffected by immune clearance and is rapidly cleared from normal tissues lacking immune suppression [92]. An et al. proposed a bacteria-based macrophage backpack, in which the attached bacteria provide powerful stimulation signals to the macrophages. Due to the tumor-homing ability of macrophages, bacteria are targeted and transported to the tumor, which further promotes the polarization of intratumor macrophages and reshapes the intratumor immune microenvironment (Fig. 1e) [93]. Coincidentally, Wu et al. created a distinct state of macrophages loaded with *Salmonella* by utilizing a shocked macrophage cell line containing VNP20009. The intracellular bacteria maintain their original biological activity, undergo delayed release, and subsequently proliferate. Employing this bacterial camouflage strategy resulted in decreased clearance of bacteria by neutrophils and significantly enhanced bacterial accumulation in tumors following systemic administration (Fig. 1e) [94].

## 4 Synthesis of genetic circuits

The synthetic biology approach facilitates the precise regulation of gene expression in bacteria, enabling them to detect multiple signals, operate circuits, and execute reactions for tasks such as imaging, diagnostics, and therapy. On the one hand, bacteria possess the ability to sense various physical and chemical changes in their environment, a crucial feature for targeting tumors and responding to external stimuli. On the other hand, the integration of modular elements like genetic circuits and logic gates within bacteria enhances their functionality, rendering them more efficient, systematic, and programmable for applications in cancer immunotherapy.

### 4.1 Signal input and gene expression triggering

External signals are conveyed to bacteria through receptors located on the plasma membrane or within the cell. These signals act indirectly via signal transduction and cascades to modulate gene expression, facilitating environmental adaptation. These signals originate from both the tumor microenvironment and supplemental therapeutic techniques, interacting with transcription factors and promoters, and constitute the basic principle of controlling the growth of engineered bacteria and exerting therapeutic effects.

**Fig. 2:**
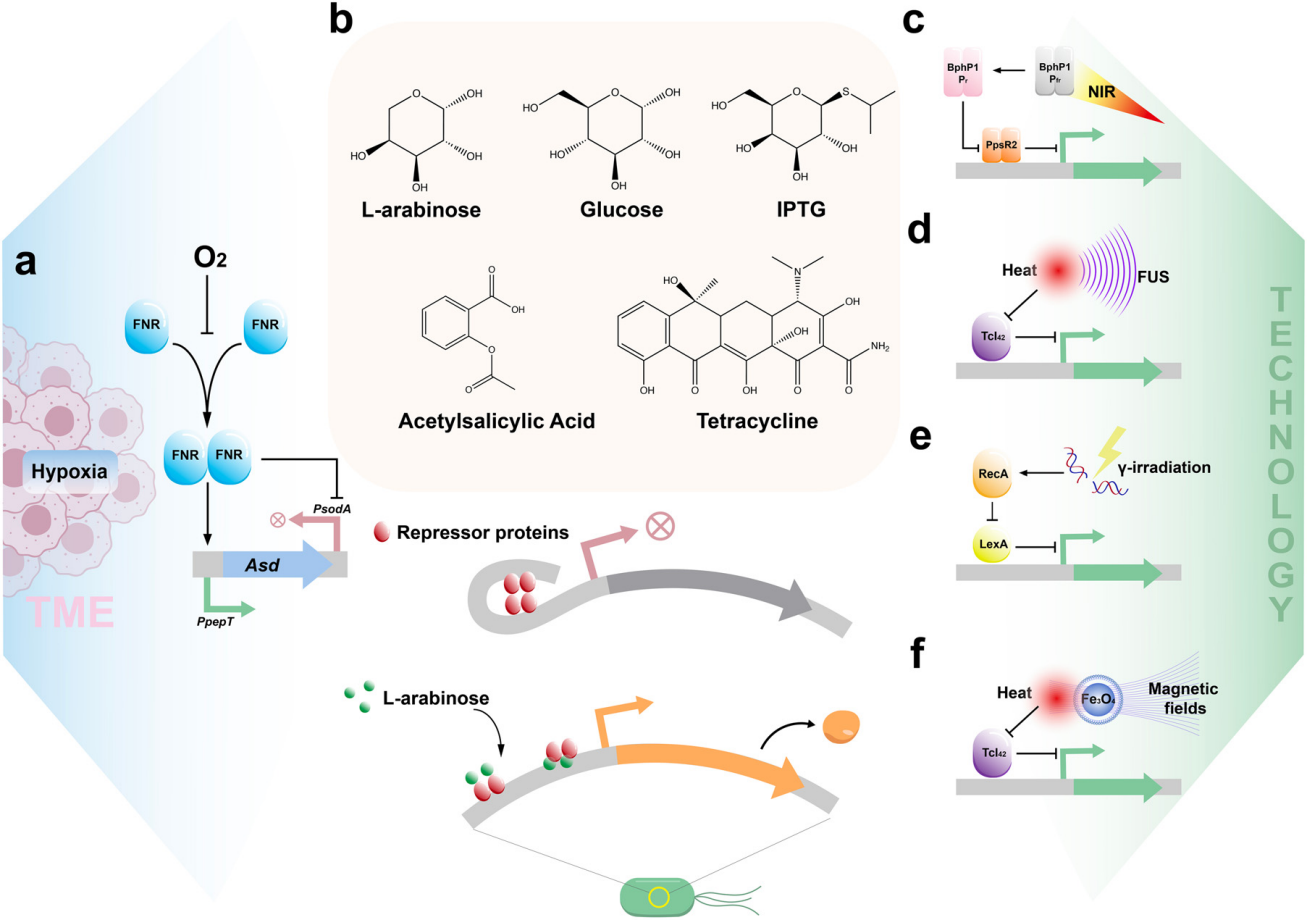
**Multiple physical and chemical signals are input and trigger gene expression.** (a) Oxygen concentration in tumor microenvironment acts as a signal to regulate bacterial growth. FNR, umarate and nitrate reduction regulatory protein, transcription factor responsive to oxygen concentration. (b) Chemical molecules that trigger gene expression can often constitute an operon model. The diagram below illustrates the principle of the arabinose manipulator. In the absence of arabinose, gene expression is inhibited. The arabinose lifts the inhibitory effect of the repressor protein on gene transcription. IPTG, Isopropyl β-d-Thiogalactoside. (c) Light signals trigger gene expression. Near-infrared light shifts BphP1 from a non-activated to an activated state, promoting gene expression by inhibiting PpsR2 [1]. (d) Ultrasound triggers gene expression. The thermal effect of focused ultrasound inhibits the function of transcription factor TcI, which initiates gene transcription [2]. (e) Radiation triggers gene expression. Ion radiation-induced DNA breaks activate RecA through a series of reactions, which regulates gene expression by inhibiting LexA [3]. (f) Magnetic field triggers gene expression. The use of a magnetic field and nanoparticles to convert the magnetic field into heat signals regulates the expression of bacterial genes [4].

Hypoxia and acidity are typical features of the tumor microenvironment, influencing bacterial targeting while serving as external input signals. As previously noted, the hypoxia-inducible promoter pPepT is regulated by the transcription activator, fumarate, and nitrate reduction regulatory protein (FNR) (Fig. 2a). Cancer cells exhibit a continual reliance on glycolysis, resulting in the release of lactic acid and the creation of an acidic tumor microenvironment [95]. Flentie et al. engineered a bioluminescent transposon reporter trap to screen a *S. typhimurium* library and identified acidic microenvironment-activated promoter sequences. By applying the STM1787 promoter to regulate the expression of Stx2 conditionally, they observed dramatic cancer cell death both *in vitro* and *in vivo* [96]. Given the crucial role of operons in prokaryotes, various chemical inducers, such as arabinose [97], glucose [98], isopropyl-beta-d-thiogalactopyranoside (IPTG) [99], salicylates [100] and antibiotics (Fig. 2b) [101]. These small molecules typically regulate the expression of associated genes through operons, making them natural candidates for synthetic genetic circuits. The advantage of chemical induction in triggering gene expression lies in its tightly regulated. Using a galactose analogue as an input signal, Danino et al. engineered an *E. coli* carrying β-galactosidase. Bacteria reach the gut by oral administration, crossing the intestinal epithelium to colonize liver tumors. By feeding LuGal, a combinatorial molecule of fluorescein and galactose, to mice, bacteria metabolize it to fluorescein. Finally, the fluorescence intensity in mouse urine reveals the tumor burden carried in the liver [98,102,103]. It poses a challenge to reduce the concentration of inducers to levels suitable for input into engineered bacteria as low-intensity signals. Exploring the design of induction factors based on the chemical characteristics of the tumor microenvironment may be instrumental in reconciling tumor targeting with the expression of genes of interest.

In addition to characteristics of the TME and exogenous chemical inducers, physical methods such as light and ultrasound can also serve as input signals to regulate gene expression in engineered bacteria. Light-regulated gene expression is the essence of optogenetics. As an ideal inducer of gene expression, light can control gene expression and cellular behavior with exceptional spatiotemporal accuracy. Photosensitive proteins are central components that enable light-regulated gene expression, converting a photon absorption event into a conformational signal. For example, bacterial phytochromes (BphPs) were described in the nonphotosynthetic bacterium *Deinoccocus radiodurans*, which are dimeric and exhibit enzymatically active effector modules [104]. Ong et al. engineered a BphP1–PpsR2 system to red-shifted optogenetic tool. Their NIR-activated transcription system does not require the production of a second messenger and exhibits rapid response dynamics (Fig. 2c) [105]. Zhu et al. developed a near-infrared light spatiotemporally responsive cancer immunotherapy platform by combining an optogenetically modified engineered bacterium EcN with lanthanide upconversion nanoparticles (UCNPs). These UCNPs attached to EcN can transform 808 nm near-infrared light into blue light, activating the blue light response system in engineered EcN. This triggers the expression and secretion of flagellin B (FlaB), which induces an immune response for tumor therapy. In summary, integrating materials technology with genetic engineering approaches offers expanded potential for light-controlled bacterial gene expression [106].

Ultrasound-based acoustic genetics is similar to optogenetic and chemogenetic techniques using optical and chemical inputs but offers the advantages of non-invasiveness, increased penetrability, and enhanced focusing ability. Biological effects induced by ultrasound encompass thermal effects, cavitation, and acoustic radiation force, enabling the manipulation of genetic switches to yield sustained therapeutic outcomes. Abedi et al. developed EcN, which expresses tumor-inhibiting nano-antibodies and features temperature-sensitive gene regulatory switches. Focused ultrasound heating to 42 °C within the tumor triggers the thermosensitive elements, leading to the expression of nanoantibodies αCTLA-4 and αPD-L1. This strategy combines targeted bacterial colonization with the localized activation of therapeutic functions by focused ultrasound, enabling synergistic therapeutic actions of bacteria and T cells from exterior to interior (Fig. 2d) [107]. Similarly, Chen et al. employed focused ultrasound to induce IFN-γ expression [108]. Although many studies have been conducted to regulate bacterial gene expression by ultrasound, methods in the form of energy with non-thermal effects have not been developed in prokaryotic cells. Developing strategies to use the mechanical effects of ultrasound as signal input remains to be investigated.

Additionally, irradiation directly penetrates tumor tissue without diffusion limitations. Ionizing radiation induces DNA breakage, activating RecA in the SOS repair system and preventing the degradation of LexA within this system (Fig. 2e) [109]. Thus, the integration of non-invasive and highly penetrating clinical technological tools facilitates precise regulation of gene expression in engineered bacteria within tumors. Magnetic genetics is a method of using magnetic fields and magnetic actuators to control biological functions. Magnetogenetics uses magnetic fields to generate force or transfer energy to magnetic actuators to perform magnetomechanical or magnetothermal stimulation of cellular targets, thereby activating intracellular pathways [110]. The advantage of magnetic genetics is that the magnetic field penetrates deep into the human body and can regulate deep tissues without intrusion, which is suitable for living applications (in contrast, optogenetics requires the implantation of light to regulate deep tissues) [111]. For micro-scale engineered bacteria, it is easy to bind to nano-magnetic actuators to regulate their gene expression. Ma et al. used iron tetroxide nanoparticles as magnetic actuators that release heat under the action of a magnetic field. The thermal effect controls the opening and closing of therapeutic genes by regulating the activity of cl protein, similar to the regulation of focused ultrasound (Fig. 2f) [112].

### 4.2 Logical expression of genes

Input signals originate from various sources, concurrently acting on engineered bacteria. Effectively receiving and processing these signals stands as a pivotal challenge in synthetic biology. To confer specific functions upon engineered bacteria, feedforward and feedback structures for signal input can be integrated within them. Memory circuits and logic gates are assembled through the concatenation, parallelization, and nesting of simple genetic circuits, enabling bacteria to perform arithmetic and output functions in response to input signals.

**Fig. 3:**
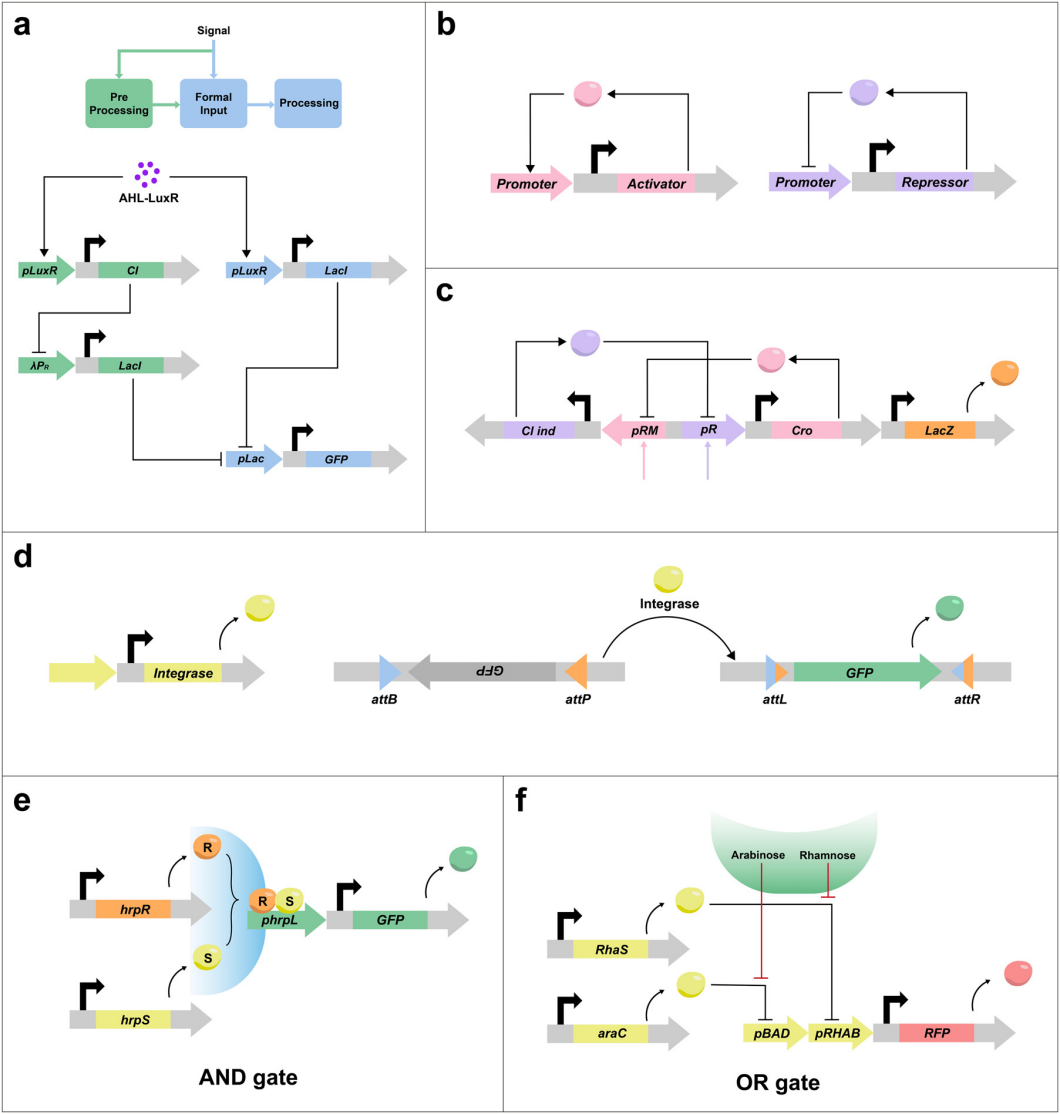
**The basic units and structures that build genetic circuits.** (a) Feedforward is the pre-processing of the input signal. The green part is the correction of the AHL concentration, which is too high or too low to end up with no GFP fluorescence signal output. AHL, acyl-homoserine lactone. LuxR, an AHL-dependent transcriptional regulator. CI, lambda repressor. LacI, lac repressor [5]. (b) Feedback effect. The products of the loop eventually backfire directly or indirectly on the loop itself. Facilitation is positive feedback (pink), and inhibition is negative feedback (purple). (c) The toggle switch is designed by connecting simple circuits in series. This conjugate structure produces a mutual inhibitory effect thereby controlling the LacZ signal output [6]. (d) The recombinases recognize the *attB* (blue) and *attP* (orange) sites and flip the internal sequence to create permanent genetic memory. (e) AND gate. HrpR and HrpS are controlled by separate promoter inputs, and the *hrpL* promoter is activated only when both genes are expressed [7]. (f) OR gate. Inhibition of the transcriptional repression of RhaS or araC by arabinose or rhamnose initiates the expression of downstream genes [8].

Feedforward refers to the signal input process, which determines the signal and provides compensation if necessary. Basu et al. constructed a feedforward circuit that effectively creates a concentration detection function for the AHL input of diffusible small molecules entering the cell. The output of the circuit responds to the AHL input only within a range of concentration values, which cannot be too low or too high (Fig. 3a) [113]. In contrast, feedback entails the system's output signal acting upon itself, categorized as positive feedback or negative feedback based on the promoting or inhibiting effect of the output signal (Fig. 3b). Positive and negative feedback are effective methods for signal conversion, amplification, and limitation. Feedback, which is employed as a strategy for regulating gene expression within the organism, enables the design of complex and diverse genetic circuits using nesting, series, and parallel methods [114]. Translation products act as transcription factors that influence the expression of other genes and participate in constructing genetic circuits. Induction and repression dictate the activation and deactivation of gene expression. Varied transcription factors and feedback effects play a crucial role in efficiently controlling the expression of reporter genes. Adhering to these principles, some memory circuits have been constructed.

The toggle switch utilizes a reverse pair of mutually inhibited inducers and inhibitors for long-time memory function and circuit state control. Comprising two repressors and two constitutive promoters, each promoter is inhibited by the repressor transcribed by the opposing promoter [115,116]. Kotula et al. constructed a toggle switch including a lambda CI/Cro-based transcriptional memory element and a tetP-Cro trigger element. This circuit is designed to start in the CI state and switch to the Cro state upon induction of a trigger element, where a tetracycline-responsive promoter controls the transcription of the *cro* gene. The stability of the CI and Cro states allows it to colonize the mouse gut effectively (Fig. 3c) [99]. Controlling the expression of recombinase to invert DNA sequences is a method of regulating gene expression. Phage recombinase recognizes the *attB* and *attP* sites, facilitating insertional recombination of DNA. Utilizing the recombinase achieves the inversion of the DNA sequence tandemly linked between attB and attP, establishing a permanent memory function (Fig. 3d) [117]. Thus, bacteria sense external signals and use synthetic genetic circuits to output therapeutic loads. This process, involving input, computation, and output, parallels a modern electronic computer, serving as a reference for designing biological systems and implementing complex controls.

Logic gates embody this concept as modularized synthetic biological components. AND gate, a fundamental logic gate circuit, performs the "with" operation, having multiple inputs and one output. The output is high (logic 1) only when all inputs are simultaneously high; otherwise, it is low (logic 0). Wang et al. constructed a modular orthogonal genetic AND gate in *E. coli* using the tightly regulated orthologous system from *P. syringae*. This AND gate integrates two independent environmental signals through hrpR/hrpS heteroregulation of σ54-dependent transcription, producing an output response in a logical manner via two transcriptional inputs (Fig. 3e) [118]. The circuit with an OR logic relationship is an OR gate, with multiple inputs and one output. If any input is high (logic 1), the output will be high (logic 1); the output is low (logic 0) only when all inputs are low (logic 0). Wong et al. utilized the restriction function of operons to construct OR gates by connecting two promoters in tandem and by inputting arabinose or rhamnose signals (Fig. 3f) [119]. Logic circuits can be constructed using DNA, RNA, transcription factors (TFs), CRISPR/Cas repressors, serine recombinases, etc. [120]. Green et al. proposed a strategy to address the challenge of evaluating complex logic in living cells by constructing RNA-only nanodevices. Their ‘ribocomputing’ system operates at the post-transcriptional level, concentrating sensing, computation, signal transduction, and output elements within a single extended RNA transcript. This design reduces signal loss, lowers metabolic costs, and enhances circuit reliability [121].

While the construction of logic gates can be intricate and variable, the task of circuit design is even more challenging. Even the assembly of simple circuits can be time-consuming and unreliable, and as circuit complexity increases, so does the difficulty of design. Electronic Design Automation (EDA) was developed for designing semiconductor electronics [122]. To simplify the creation of genetic circuits, Nielsen et al. applied EDA principles. They developed a design environment, called Cello, in which user-written Verilog code describes circuit functions and is automatically translated into DNA sequences. The algorithm then constructs the circuit diagram, assigns and connects the gates, and simulates performance. This approach of using automation principles to design circuits in bulk and screen efficient solutions has significantly enhanced the efficiency of engineering bacterial modifications [123].

## 5 Payload delivery and release

In experiments, bacteria are generally injected directly into the tumor to obtain a better therapeutic effect, and it is safer than intravenous injection. Orally delivered bacteria overcome the intestinal barrier and escape to the circulatory system. As living organisms, unlike other non-living carriers or drugs, bacteria can overcome entropy increase and thus generate activities to cope with the complex environment and actively seek out tumors *in vivo* [12]. Ideally, bacteria are initially distributed in low doses to blood vessels throughout the body, with the abundance in the tumor gradually increased by the anoxic tumor microenvironment and targeted strategies. Any bacteria remaining in the bloodstream are cleared by the immune system. However, practical applications may encounter challenges such as inflammation and bacterial enrichment in normal organs. The controlled delivery and release of drugs by successfully colonized bacteria directly impact immunotherapy efficacy. Effective methods of drug release include secretory action, bacterial lysis, and membrane-based delivery.

### 5.1 Direct secretion of therapeutic molecules

**Fig. 4:**
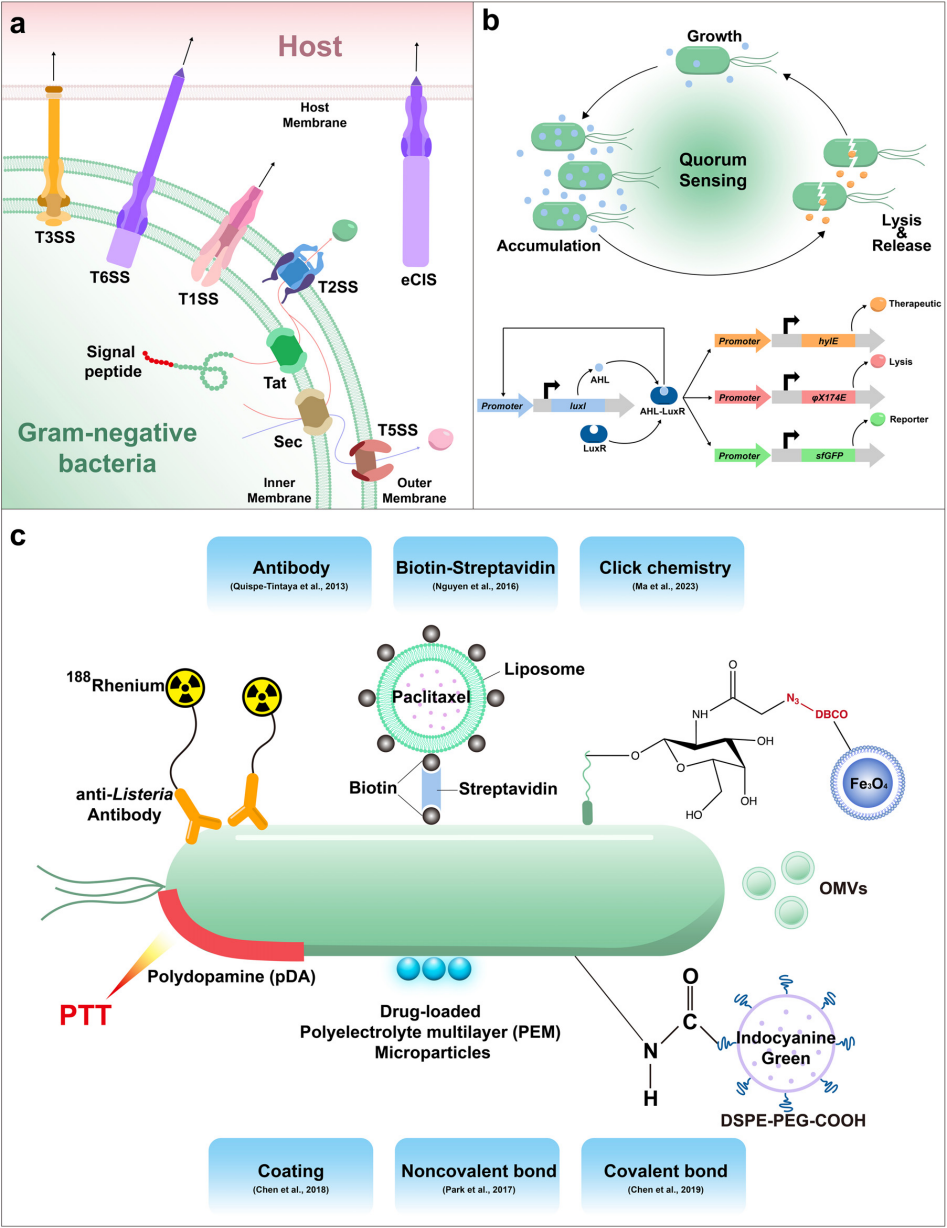
**Mechanisms of drug loading and release by engineered bacteria.** (a) Protein secretion system of gram-negative bacteria. Signal peptides are critical for directing protein secretion. (b) The quorum sensing circuit realizes the periodic change of bacterial population, which leads to stable and sustained drug release. With the increase of the population, AHL induces the expression of lytic and drug proteins, causing bacteria to lyse and enter the next cycle. φX174 E, a bacteriophage lysis protein. HlyE, a pore-forming anti-tumor toxin [9]. (c) Multiple drug loading strategies of bacterial cell membranes.

Bacteria deploy diverse secretion systems to transport proteins into the cytoplasmic matrix or cytoplasm of the host through specialized secretory mechanisms (Fig. 4a) [124]. The general secretion (Sec) and twin arginine translocation (Tat) pathways are the primary bacterial systems for transporting proteins across the cytoplasmic membrane [125]. Both systems are found in gram-negative and positive bacteria, whose primary role is to secrete proteins outside the cell membrane. Sec pathway transports proteins in an unfolded state, while Tat pathway secretes proteins in a folded state [126]. Proteins delivered by the Sec or Tat system remain in the periplasm, and some will ultimately become extracellular. Once in the periplasm, they can be transported across the outer membrane with the assistance of the T2SS and T5SS [127]. Signal sequences, such as PelB [128], OmpA [129], and DsbA [130], are required for proper guidance of protein secretion to the extracellular space. The signaling sequence directs the secretion of functional proteins into the tumor extracellular matrix, releasing the therapeutic payload [131,132].

Another mode of secretion involves bacteria injecting proteins directly into the host cytoplasm. Several protein secretion systems in gram-negative bacteria can transport substrates across both membranes in a single step, independent of the Sec or Tat pathways. These systems include T1SSs (type I secretion systems), T3SSs, T4SSs, T6SSs, and others. Each of these pathways forms channels that span the periplasm and transport proteins from the cytoplasm to the extracellular environment, though they utilize different mechanisms for protein secretion. Notably, T3SSs, T4SSs, and T6SSs can also translocate proteins across an additional host cell membrane, delivering them directly into the cytosol of the target cell [127]. Taking the example of type III secretion systems (T3SSs), which have entered medical applications, bacteria initially make membrane contact with the target cell. The bacteria then deliver transposons to the target cell via the secretion complex on the bacterial membrane, perforating the plasma membrane of the target cell and forming a channel [133]. T6SSs share similarities with T3SSs and are another bacterial secretion system with medical implications [134]. In synthetic biology, controlling the expression of protein complexes in microorganisms for biomedical applications presents a significant challenge. While T3SS protein delivery systems can inject proteins into mammalian cells, transferring them to non-pathogenic bacteria has proven difficult. To address this issue, Ruano-Gallego et al. successfully assembled functional injectisomes from the T3SS of pathogenic *E. coli* in non-pathogenic *E. coli* K-12, achieving protein injection into mammalian cells. Their modular structure allows for the exchange of promoters for specific applications, thereby enhancing the flexibility of the engineered bacteria [135]. T6SSs exhibit structural homology to phage tails, leading to the hypothesis that T6SSs may have evolved from inverted phage tails ejecting proteins outside of the bacterial cell rather than injecting them inside the cell [136]. Extracellular contractile injection systems (eCISs), considered close relatives of T6SSs, resemble headless phages, being released into the medium and binding to the target cell surface [137]. Kreitz et al. demonstrated that eCISs function as programmable protein delivery devices depending on the tail fiber's binding to a receptor on the target cell. They can be modified to load non-native payloads and target novel organisms [138]. Their efforts are expected to enhance the efficiency of protein delivery by bacteria, thereby improving the effectiveness of immunotherapy. Given the distinct gene expression processes in prokaryotic and eukaryotic cells, proteins expressed by bacteria may exhibit defects in folding and modification, compromising normal functionality.

### 5.2 Bacteria lysis-induced release of contents

Drug release is closely related to the number of bacteria and the degree of lysis. While bacterial lysis is a prerequisite for drug release, complete bacterial lysis is counterproductive to achieving sustained therapeutic effects, resulting in weak tumor suppression. Consistent drug efficiency is influenced by the continuous and stable presence of microbiota and controllable bacterial lysis. Integrating genes associated with bacterial lysis in the design of genetic circuits is a sensible approach to controlling bacterial populations. The effective regulation of bacterial lysis is achieved through the design of genetic circuits and the modulation of gene expression using various inducers. Bacterial quorum sensing is a strategy for regulating gene expression that orchestrates collective group behavior [139]. Danino et al. described an engineered gene network featuring global intercellular coupling, capable of producing synchronized oscillations in a growing cell population [140]. To maintain bacterial populations at a periodical-changed level and sustained drug release, a quorum-sensing gene circuit was designed for synchronized control of bacterial populations and repeated cycles of therapeutic payload release. Based on feedback principles, this circuit includes a promoter that drives the expression of its own activator and a lysis gene. Specifically, the *luxI* promoter regulates the production of AHL autoinducer, which binds LuxR and activates the promoter transcriptionally. Cell death is triggered by a bacteriophage lysis gene (*φX174 E*), also controlled by the *luxI* promoter. AHL diffusion to neighboring cells serves as an intercellular synchronization mechanism (Fig. 4b) [141]. When the number of bacteria is low, the bacteriophage ϕX174 protein E is secreted extracellularly and at low concentrations, facilitating bacterial colonization. As the bacterial count increases, protein accumulation leads to bacterial lysis and subsequent drug release, regulating the bacterial population in a cyclical manner and ensuring sustained efficacy.

### 5.3 Transport of particles and bacteria-derived membrane vesicles

Bacteria have been utilized to enhance the capabilities of biohybrid microswimmers designed to transport synthetic vehicles, such as liposomes, nanoparticles, and hydrogels, for the propulsion, guidance, and delivery of diverse drug cargoes (Fig. 4d) [142,143]. Previous investigations have explored the use of bacteria as carriers for delivering particles essential for radiotherapy [144], chemotherapy [145] and photothermal therapy (PTT) [146], highlighting the potential of bacterial delivery systems in facilitating the combination of immunotherapy with other cancer treatment modalities. Ektate et al. attached synthetic liposomes to the surface of *Salmonella* by biotin-streptavidin. The chemotherapeutic drug was released by focused ultrasound, which enabled the combination of chemotherapy and immunotherapy [147]. Park et al. utilized surface charge and noncovalent interactions to bind *E. coli* to polyelectrolyte multilayer (PEM) microparticles encapsulating the chemotherapeutic drug doxorubicin (DOX) and magnetite (Fe_3_O_4_) nanoparticles (MNPs). By testing targeted drug delivery in an *in vitro* 4T1 breast cancer cell model, it was demonstrated that their microswimmer design was able to encapsulate cancer drug molecules in PEM carriers to target breast cancer cells efficiently [148]. PTT works by absorbing light energy using photothermal agents such as gold/iron oxide and graphene-based nanomaterials, which are then converted to thermal energy upon exposure to light irradiation [149]. Compared with widely used photothermal agents, melanin-like polydopamine (pDA) shows better biocompatibility and potential for photothermal therapy due to its excellent biodegradability. By preparing pDA-coated VNP20009 via dopamine oxidation and self-polymerization, Chen et al. managed to achieve tumor targeting and tumor elimination without relapse or metastasis with only one injection combined with laser irradiation [150]. Chen et al. attached nano-photosensitizers (indocyanine green (ICG) -loaded nanoparticles, INPs) to the surface of YB1 covalently through amide bonds to form biotic/abiotic crosslinked systems (YB1-INPs) for precision cancer therapy. YB1 remains viable after efficient linking to INPs [146]. Ma et al. used click chemistry to site-specifically label bacteria with Fe_3_O_4_ nanoparticles in response to a magnetic field and produce temperature changes thereby regulating gene expression [112]. This association of nanoparticles with engineered bacteria realizes the linkage of material modification and gene modification, which dramatically expands the functions and applications of engineered bacteria.

Furthermore, membrane vesicles derived from bacteria represent another method for drug delivery and release. Membrane vesicles (MVs) are phospholipid bilayer particles with a diameter of 20 to 400 nm that are secreted during bacterial growth [151]. MVs are associated with various biological processes in bacteria, such as virulence, horizontal gene transfer, cell metabolite export and cell communication. Outer membrane vesicles (OMVs), natural vesicles secreted by gram-negative bacteria, activate the innate immune system and engage with the host immune system due to their abundant pathogen-associated molecular patterns (PAMPs) [152]. OMVs exhibit the capability to traverse the intestinal epithelial barrier, interacting with immune cells in the lamina propria, particularly dendritic cells (DCs), and inducing immune regulation [153,154]. Cheng et al. display antigens on the OMV surface to stimulate specific anti-tumor immune responses. The tumor antigen is displayed on the OMV surface by fusion with ClyA protein, and then the antigen presentation process is simplified by using a tag/catcher protein. It also shows that many different tumor antigens can synergistically stimulate anti-tumor immune response. In addition, bioengineered OMV loaded with varying antigens of tumor inhibited pulmonary melanoma metastasis and subcutaneous colorectal cancer growth. Their platform based on bioengineered OMV could facilitate the development of personalized tumor vaccines [155]. Surface coating of OMVs not only diminishes their immunogenicity, significantly increasing the safely administered intravenous dose but also equips the formulation with radiation-triggered controlled release of CD47 nanobody [156]. Since OMVs are critical for the bacterial-host immune interaction, it is crucial to engineer engineered bacteria to produce OMVs with therapeutic loading for dose reduction continuously. The use of OMVs as mediators of interactions between bacteria and the host immune system has profound research implications and clinical translational value.

## 6 Cancer immunotherapy strategies

The bacterial immunogenicity initially triggers the immune system via Toll-like receptors (TLRs), eliciting robust innate and adaptive immune responses. Engineered bacteria also harbor immunomodulatory capabilities, including cytokine expression, which augments immune cell activation and enhances antitumor efficacy. Moreover, these vectors facilitate the precise delivery of immune checkpoint inhibitors, such as CTLA-4 and PD-1/PD-L1 antibodies, to TME, thereby restoring T cell functionality and fostering potent antitumor immune responses. Additionally, they serve as efficient carriers for tumor vaccines, delivering cancer-specific antigens to elicit adaptive immune responses, consequently resulting in tumor regression and mitigating tumor recurrence (Table 2).

**Table 2:** **Engineered bacteria for cancer immunotherapy**.

Strategies	Effectors	Refs
Immunogenic molecules	LPS (endogenous)	[158,161]
	Engineered flagellin (FlaB)	[131,177]
	CpG DNA	[195]
Cytokines	IL-2	[171,48,205,206,207,172]
	IL-4	[208]
	IL-12	[209,175]
	IL-15	[177]
	IL-18	[210,208,178]
	TNF-α	[211,109]
	LIGHT	[181]
	IFN-γ	[212]
	GM-CSF	[209]
	FLT3L	[213]
	CCL21	[182]
Immune checkpoint inhibitors	Anti-CTLA-4 nanobody	[194,107]
	Anti-PD-L1 nanobody	[194,107]
	PD-L1 siRNA	[195]
	CD47 nanobody	[214,156]
Vaccines	NY-ESO-1 tumor antigen	[197]
	Tetanus toxin protein	[198]
	Tumor antigens in situ	[199]
	Antigen-bearing OMVs	[200]
	DNA vaccine	[204]

### 6.1 Immunogenic bacteria activate the immune response

**Fig. 5:**
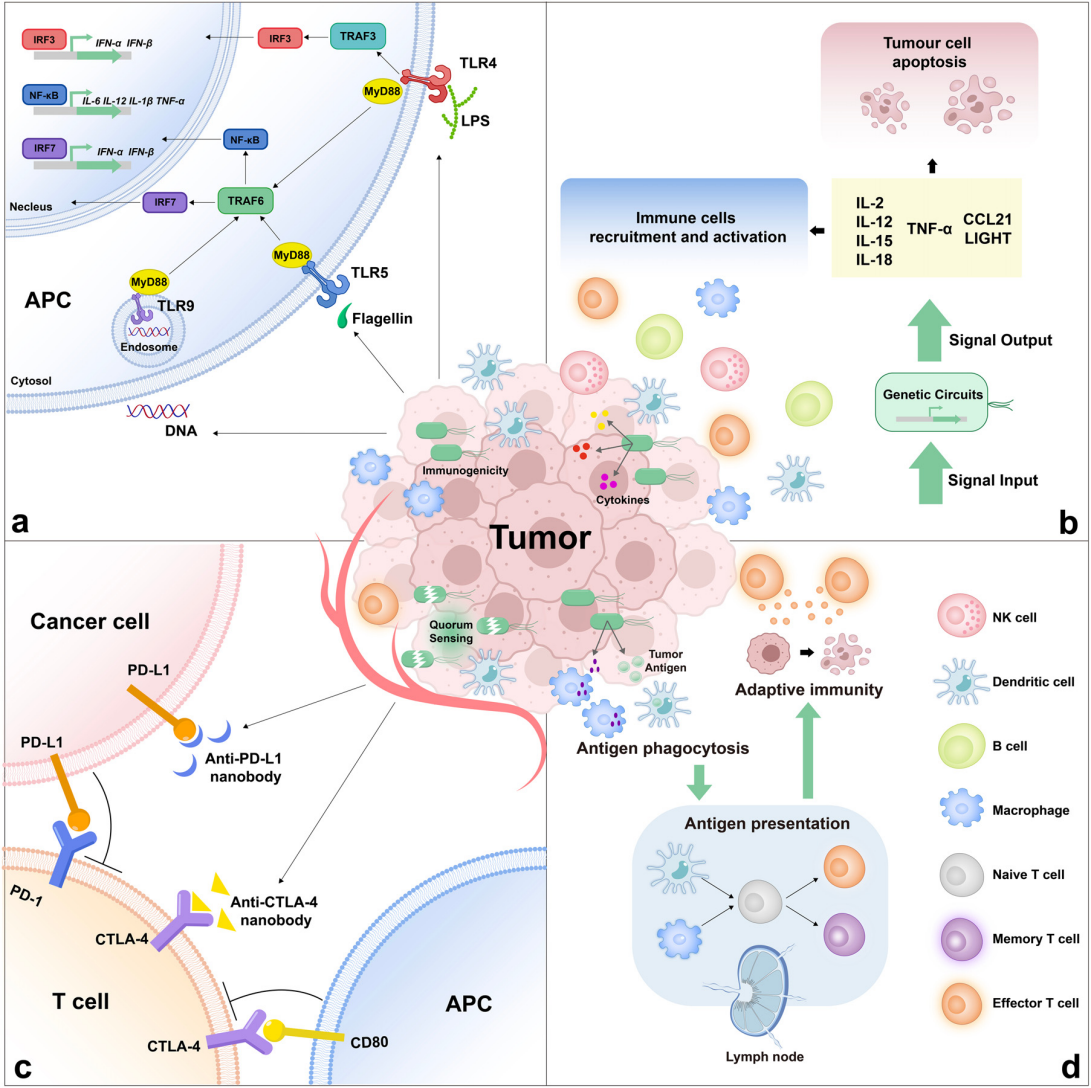
**Principles of cancer immunotherapy utilizing engineered bacteria.** (a) Immunogenicity of bacteria. Bacterial components activate Toll-like receptors that elicit the expression of anticancer cytokines through signal transduction. (b) The engineered bacteria express a variety of immunomodulatory factors. Synthetic gene circuits endow the engineered bacteria with the function of expressing cytokines and chemokines, causing immune cell activation and tumor cell apoptosis. (c) Immune checkpoint inhibitors. The quorum sensing circuit is used to lyse bacteria and stably release immune checkpoint inhibitors to regulate T cell activity [10]. (d) Cancer vaccines. After the phagocytosis and presentation of tumor antigens carried by the engineered bacteria by macrophages and dendritic cells, the effector T cells are activated to kill cancer cells, and the resulting memory T cells play a long-term anti-tumor function.

Bacteria inherently possess immunogenic characteristics stemming from the activation of innate immune receptors through the expression of surface and intracellular biomolecules. They secrete immunostimulatory metabolites and effector proteins, facilitating infiltration into tumors and local immune cells. When bacteria colonize a tumor, their outer membrane ligands trigger the recruitment and activation of monocytes, macrophages, and neutrophils, promoting an innate immune response. Antigen-presenting cells (APCs) then enter the tumor, phagocytose dead tumor cells and intratumoral bacteria, thereby boosting the antitumor response (Fig. 5a) [157]. Microbe-associated molecular patterns (MAMPs) trigger signaling cascades by binding to surface pattern recognition receptors (PRRs), which can lead to bacterial pathogenesis. An example is toll-like receptor 4 (TLR4), which detects lipopolysaccharides (LPS) on the outer membrane of gram-negative bacteria [158]. TLRs signaling pathway activation induces the upregulation of genes encoding type I interferons and cytokines (e.g., TNF-α, IL-1, and IL-6), fostering anticancer effects and inflammation [159]. TLRs promote the immunostimulatory potential of DCs through the MyD88-dependent signal pathway and the TRIF-dependent signal pathways [160]. TLRs also play an essential role in DC maturation, stimulating macrophages, dendritic cells, and other APCs to produce pro-inflammatory substances, cytokines, chemokines, and their receptors, which initiate antigen-specific adaptive immune responses [161].

Hence, the LPS-TLR4 interaction triggers the activation of innate and adaptive immunity, constituting the initial response to bacteria-based cancer immunotherapy. However, the scope of cancer treatment relying solely on the LPS-TLR4 pathway is limited, as evidenced by the inhibitory effect of purified LPS against CT26 tumors being comparable to live bacteria-mediated immunotherapy but less effective against other tumors [162]. Flagellin targets TLR5, with downstream signaling pathways interacting with LPS-induced antitumor mechanisms, leading to the secretion of anti-tumor effectors and an enhanced tumor suppressive effect. Engineered *Salmonella* expressing and secreting heterologous flagellin (FlaB) exhibits superior antitumor effects. The combined action of LPS and exogenous FlaB activates TLR4 and TLR5 signaling pathways, inducing monocyte, macrophage, and neutrophil infiltration into the tumor microenvironment. FlaB secretion activates intratumoral macrophages with M1 phenotypes, resulting in an anti-tumor effect and a reciprocal reduction in M2-like suppressive activities. The synergistic function of these two components suggests that bacteria are immunogenic and that engineered bacteria can encode and secrete payloads, leading to cancer immunotherapy [131]. This approach suggests that some of the characteristics of the causative organisms could be fused to a safe delivery vehicle, which would exert a potent anti-tumor effect without serious adverse effects. Beyond surface structural features, bacterial genetic material acts as a ligand activating TLR signaling. TLR9 recognizes bacterial unmethylated CpG DNA motifs, activating downstream TLR9-MyD88-NF-κB and cGAS-STING pathways, enhancing cytokine release (including type I IFN, TNF-α and IL-6), and consequently activating the host immune response [[163], [164], [165], [166]].

### 6.2 Expression of multi-layered immunomodulatory functions of cytokines

In addition to eliciting an immune response, bacteria produce cytokines that activate the host immune system, eliminating cancer cells through the upregulation of immune cell activation, proliferation, and migration (Fig. 5b). Interleukin-2 (IL-2), a pleiotropic cytokine generated post-antigen activation, plays a crucial role in the immune response [167]. IL-2 exerts diverse pleiotropic actions, including promoting T cell proliferation, survival, cytolytic activity, NK cell activity, Treg cell development, and activation-induced cell death (AICD), with minimal side effects, making it a potential localized treatment for various cancers [[167], [168], [169], [170]]. Studies indicated that IL-2-expressing *Salmonella* and *E. coli* modulate the tumor microenvironment, suppressing malignancy [171,172]. Interleukin-12(IL-12) is a potent antitumor cytokine with a variety of antitumor properties, but its clinical use is limited due to the toxicity of systemic administration [173,174]. The genetically engineered *Clostridium sporogenes*, which can secrete IL-12, can selectively settle and reproduce in tumors after intravenous injection and significantly inhibit tumor growth. Accurate targeting and local release of IL-12 may explain why this therapy does not elicit a systemic response [175]. As a promising agent for anticancer immunotherapy, IL-15 is implicated in the processes of development, maturation, and activation of CD8^+^ T cells, NK cells, and NKT cells, alongside its role in regulating the survival and proliferation of CD8^+^ T memory cells [176]. Zhang et al. reported on an attenuated strain of *S. typhimurium* that produced a fusion protein combining FlaB and IL-15, synergistically enhancing both anti-tumor activity and long-term immune memory [177]. In addition, nonvirulent *Salmonella* engineered to synthesize the cytokine IL-18 enhanced antitumor activity in preclinical mouse cancer models. IL-18-producing bacteria inhibited the growth of primary subcutaneous tumors and pulmonary metastases without overt toxicity to normal tissues [178]. TNF-α is a crucial component of both innate and acquired immunity, capable of inducing apoptosis in tumor-associated cells, resulting in tumor cell destruction [179]. Salmonella carrying a prokaryotic expression vector encoding TNF-α demonstrated activity as a tumor-targeting anticancer agent and adjuvant in syngeneic murine tumor models [180]. Cytokine LIGHT and chemokine CCL21 can induce inflammatory cell infiltration, exerting anti-tumor effects. Attenuated Salmonella expressing CCL21 or LIGHT significantly inhibited the growth of primary tumors and lung metastases, accompanied by increased levels of IFN-γ, CXCL9, and CXCL10 in mouse tumors [181,182].

### 6.3 Delivering immune checkpoint inhibitors to tumor

The immune checkpoint pathway regulates immune responses through ligand/receptor interactions, crucial for maintaining autoimmune tolerance and controlling the duration and strength of the immune response. This regulation helps prevent immune system-mediated damage to normal tissues [183]. Essentially, the immune checkpoint serves as a braking mechanism to prevent overstimulation of the organism's immune system. Tumor cells exploit this ‘brake’ mechanism to suppress immune cell function. Consequently, blocking the molecular interaction becomes the central principle of this therapeutic approach. Cytotoxic T lymphocyte-associated protein 4 (CTLA-4) has demonstrated potent inhibitory effects on regulating T cell responses [184]. Generally localized to intracellular vesicles, CTLA-4 translocates to the cell surface upon activation of T cells by the interaction of MHC and BF7 (CD80 and CD86) in antigen-presenting cells with TCR and CD28 in T cells. This translocation allows CTLA-4 to bind to CD28 competitively, inhibiting the arrest of T cell proliferation and activation [183]. Programmed cell death protein 1 (PD-1), expressed in various immune cells like activated T cells, B cells, and natural killer (NK) cells [185], suppresses the immune response when interacting with its ligands PD-L1 or PD-L2 [186,187]. Cancer cells express antigens that inhibit immune cell activity, evading surveillance and clearance by the immune system [188]. The expression patterns of PD-L1 and PD-L2 vary across tumor types [189], and their interaction with PD-1 on tumor-infiltrating lymphocytes (TILs) is suggested as a mechanism of tumor immune escape. Blocking this pathway holds the potential for less severe immunotoxicity than CTLA-4 blockade [190]. Inhibiting PD-1 and CTLA-4 allows T-cell activation, restoring antitumor activity and immune function [191]. Immune checkpoint inhibitors give great impetus to cancer immunotherapy, achieving tumor regression in various cancers [192,193]. As previously described for the quorum-sensing circuit, Gurbatri et al. designed engineered bacteria to control the production and release of PD-L1 and CTLA-4 nanobodies. Of interest, the authors simulated the amount of therapeutic protein by varying the copy number of the gene as a way of probing the kinetic parameters of the circuit and optimizing therapeutic strategies. Their proposed probiotic system resulted in tumor clearance and prolonged survival, with a single injection inducing an adaptive immune system-mediated durable therapeutic response compared to animals treated with antibody combinations (Fig. 5c) [194]. Similarly, Abedi et al. used focused ultrasound to heat the tumor to 42 °C to trigger nanoantibody αCTLA-4 and αPD-L1 gene expression. This strategy directly regulates the expression and release of engineered bacterial therapeutic proteins in tumors and avoids the risk of load leakage [107]. Targeting PD-1 via siRNA delivery, combined with CpG ODN, showed anti-tumor effects when delivered by attenuated *Salmonella*, inhibiting melanoma and enhancing antitumor immune responses in mice [195]. Currently, relatively few studies have been conducted on the delivery of immune checkpoint inhibitors via engineered bacteria. As a kind of living delivery carrier, engineered bacteria can penetrate deeply into the tumor, which is expected to overcome the shortcomings of traditional immune checkpoint inhibitors, such as poor targeting and low concentration, and release active proteins efficiently in the tumor to restore the function of the immune system.

### 6.4 Tumor vaccines

Tumor vaccines serve as a proactive method for cancer prevention. Delivery of cancer-specific antigens, antibodies, growth factor-targeting domains, and proteins aimed at anti-apoptosis or tumor-associated macrophages by bacteria stimulates immune responses, promotes inflammation, and improves T cell antigen presentation (Fig. 5d) [196]. A vaccine strain of *S. typhimurium* delivers the NY-ESO-1 tumor antigen via the type III protein secretion system. Intratumoral inoculation of *S. typhimurium*–NY-ESO-1 in NY-ESO-1–negative tumors resulted in antigen delivery, leading to tumor regression in the presence of preexisting NY-ESO-1–specific CD8^+^ T cells [197]. Tetanus is a severe disease caused by a toxic protein secreted by *Clostridium*. Tetanus vaccine-induced memory T cells specific to tetanus circulate in the bloodstream for life. Individuals vaccinated against tetanus mount a robust immune response upon subsequent exposure to highly foreign tetanus toxins. *Listeria monocytogenes* carrying tetanus toxin-encoding gene, infect tumor cells and express the tetanus toxin protein within them, triggering an anti-tumor immune response. This mechanism utilizes the activation of preexisting tetanus-specific memory T cells, causing CD4^+^ T cells to attack and eliminate the infected tumor cells [198]. Wang et al. implemented tumor vaccine therapy in situ using engineered bacteria. They injected nanoparticle-modified *Salmonella* into tumors after radiotherapy. Antigens generated by radiotherapy were captured by this bacteria and actively moved into the tumor margin tissue for antigen presentation, which increases systemic antitumor immune responses after radiotherapy [199]. Under regulation by an arabinose-inducible promoter, engineered *E. coli* produced outer membrane vesicles (OMVs) containing cytolysin A tumor antigen and the Fc fragment of mouse IgG. These OMVs efficiently traversed the intestinal epithelium to reach the lamina propria, inducing dendritic cell maturation. In mouse models of metastatic melanoma and subcutaneous colon tumors, these antigen-loaded OMVs suppressed tumor growth and conferred protection against tumor re-challenge [200]. This vaccine strain releases tumor antigens in the gut through oral administration, improving the safety of treatment. DNA vaccines against cancer offer advantages over conventional therapies, being cost-effective and exhibiting prolonged memory in the body [201]. Such vaccines deliver the DNA sequence to trigger innate immune responses by the cGAS-STING signaling pathway, and its encoded therapeutic protein efficiently induces both the humoral and cell-mediated immune responses [202]. DNA vaccines have been shown to impede the progression of tumors by triggering T-cell and antibody immune responses against tumor self-antigens [203]. When delivered by attenuated *S. typhimurium*, an MTDH/AEG-1-based DNA vaccine induces robust CD8^+^ cytotoxic-T-cell-mediated immune responses against breast cancer, displaying effectiveness in preventing tumor growth and metastasis [204].

## 7 Discussion and conclusion

In cancer immunotherapy, the utilization of engineered bacteria has emerged as a promising avenue. The engineering methods employed for ensuring safety in this context constitute a crucial foundation. Diverse strains, with bacterial native anticancer effects, have toxic side effects on the host. As a fundamental objective, the reduction of toxicity is achieved through the application of genetic mutation. To enhance the precision of cancer treatment, tumor-targeting strategies are devised, involving microenvironment-mediated bacterial orientation and molecular interactions amplifying specific tumor targeting. Assistant methods based on advanced technology and specific immune cells are also employed to deliver bacteria, ensuring targeted therapeutic impact efficiently. The synthesis of genetic circuits is central to the innovation of these therapeutic bacteria. Controllable triggering approaches and the logical expression of genes contribute to the development of sophisticated and responsive genetic systems. Efficient payload delivery and release mechanisms further determine therapeutic efficacy, encompassing diverse strategies such as direct secretion of therapeutic molecules, bacteria lysis-induced release of contents, particle transport, and bacteria-derived membrane vesicles. Engineered bacteria-based cancer immunotherapy embraces a multifaceted approach, with immunogenic bacteria activating the immune response and the expression of multi-layered immunomodulatory functions of cytokines, which help overcome the limitations of cancer treatment. Delivering immune checkpoint inhibitors directly to the tumor and the development of tumor vaccines also contribute to the clinical translation of cancer immunotherapy.

Although the clinical application of bacteria as cancer immunotherapy carriers has shown great potential, it also comes with some important challenges. It mainly includes the risk of uncontrolled bacterial infection caused by individual differences of patients, recovery of virulence caused by bacterial gene mutation, interference with patients' microbiome, pharmacodynamic and pharmacokinetic characterization methods, and difficulties in drug scale production.

A major risk of bacterial therapy is that uncontrolled bacterial proliferation can trigger systemic or local infections, especially if the patient's immune function is suppressed. Although the bacteria usually target tumor tissue, their spread and proliferation in the host is not always controllable. Once the bacteria escape into healthy tissues outside the tumor, it can lead to serious infection or sepsis, even life-threatening [215]. Bacteria are genetically engineered to have exogenous control switches. For example, bacteria are engineered to respond to specific external signals, thereby controlling their proliferation and activity when needed [216]. This technique has been used to design bacteria that are sensitive to small molecules such as arabinose, capable of activating or inhibiting bacterial proliferation by applying an external signal. It is also possible to construct "self-limiting" bacteria by introducing a time-control mechanism or self-destruct switch to ensure that the bacteria automatically die under certain time or metabolic conditions, thereby preventing their unlimited spread [141]. This can be achieved by regulating key metabolic genes in the bacteria, and once the preset environment is exceeded, the bacteria will automatically lose the ability to survive.

Bacteria that are genetically modified often weaken or lose their virulence, making them safe for clinical use. However, there is a risk of bacterial gene mutation or horizontal gene transfer promoting bacterial virulence relapse, which may lead to its resumption of pathogenicity, thus posing a serious threat to patient health [217]. Especially in immunosuppressed patients, virulence recovery can lead to serious infection. To minimize the possibility of virulence recurrence, multiple genes associated with virulence can be deleted by multiple gene knockout or genomic modification. This multi-site modification can reduce the probability of mutation restoration of pathogenicity. For example, permanent gene knockout targeting multiple virulence genes in bacteria, while deleting genes associated with biofilm formation, makes the bacteria easier to be cleared by the host immune system. Another approach is to use synthetic biology to design switches that can sense the environment and, once the expression of virulence genes is detected, turn on and inhibit their function or directly induce bacterial death. Through this negative feedback mechanism, the recovery of virulence can be effectively avoided [218].

The introduction of exogenous bacteria into bacterial therapy may have an impact on the host's microbiome, resulting in an imbalance in the host's microecosystem. This imbalance may lead to a series of side effects, such as gastrointestinal discomfort caused by intestinal flora disturbance or immune dysfunction [219]. Microecological imbalance may also increase the risk of drug-resistant bacteria, which in turn affects the overall treatment effectiveness. By improving the delivery system of bacteria and enhancing their targeting, their impact on the microbiome of healthy tissues can be reduced. Highly targeted bacterial therapy will ensure that bacteria mainly play a role in the tumor microenvironment and reduce their colonization in other tissues, thereby reducing interference with the systemic microbiome. In order to maintain the microecological balance of the host, probiotics or other symbiotic bacteria can be combined with bacterial therapy. These beneficial bacteria can help maintain the normal microbial structure of the host, thereby mitigating the negative impact that exogenous bacteria may have on the host microbiota during treatment [220].

As a drug delivery vehicle in cancer immunotherapy, the characterization and quantification of pharmacodynamics (PD) and pharmacokinetics (PK) of bacteria are crucial to optimize the therapeutic effect. Unlike traditional medicines, bacterial therapies involve the complex metabolism and mechanisms of action of living cells, so new characterization methods and quantitative indicators need to be developed for their unique biological characteristics. In terms of pharmacodynamic characterization, the core of bacterial therapy is its interaction with the tumor microenvironment and activation of the immune system. After the bacteria enter the body, they achieve local drug release and immune activation by targeting hypoxic or necrotic areas of the tumor. Therefore, the key indicators to quantify its pharmacodynamic effect include the ability of bacteria to colonize the tumor site, the level of local cytokine release, the degree of activation of antigen-presenting cells, the infiltration and activation of T cells, and the inhibition or regression of tumors [221]. Quantification of these immune and tumor responses often relies on *in vivo* imaging techniques (such as *in vivo* fluorescence imaging and bioluminescence imaging), immunohistochemical analysis, and flow cytometry. In addition, genetic modification of bacteria can be used to precisely control their drug release kinetics and monitor their activity in real time by introducing reporter genes. In terms of pharmacokinetic characterization, the metabolic pathway of bacteria is different from that of traditional chemical drugs, and the process of distribution, proliferation and clearance *in vivo* is more complicated. Therefore, its PK characteristics need to be quantified from multiple dimensions. Firstly, the biological distribution of bacteria in the host is the primary link of pharmacokinetic characterization. Studies have shown that the colonization ability and migration rate of bacteria in different organs and tumor tissues are affected by various factors [12]. Real-time tracking of the distribution of bacteria in the body can be achieved through molecular imaging techniques (such as positron emission tomography, PET), which can monitor the dynamic behavior of bacteria in the body with high precision [222]. Secondly, the rate of bacterial proliferation and clearance is the key index of its pharmacokinetics. Unlike the simple metabolism of traditional drugs, bacteria can self-proliferate in the body and may be eliminated by recognition by the immune system. Therefore, it is crucial to evaluate its proliferation rate, bacterial load in blood or tissues, and immune-mediated clearance time [223]. These quantifications can be accomplished by quantitative culture of bacterial populations, PCR analysis, or fluorescence reporting systems to derive survival curves at different time points. In addition, the pathway of bacterial clearance, such as by immune clearance of the host or natural death, must be quantitatively described by the persistence of the bacteria in different organs of the host and the response of the immune system. In summary, the characterization and quantification of the pharmacodynamics and pharmacokinetics of bacteria as drug delivery vehicles involves multi-dimensional considerations ranging from the colonization, proliferation, metabolism and clearance processes of bacteria in the body to the response of the immune system to them. Through advanced imaging techniques, immunological analysis methods and genetic engineering modification, systematic PD and PK models of bacterial therapy can be gradually established. This not only contributes to a deeper understanding of the mechanism of action of bacterial vectors, but also provides a quantitative basis for clinical evaluation of their safety and efficacy.

In the large-scale manufacturing and circulation of engineered bacterial drugs, large-scale production and purification technology, quality control means, and transportation and preservation are three key links. First, the large-scale culture and fermentation production of bacteria requires precise control of growth conditions, such as temperature, pH and oxygen concentration, to ensure bacterial activity and genetic stability. This process must optimize the fermentation process and use advanced bioreactors to ensure efficient bacterial proliferation and functional preservation [224]. In addition, bacterial cultures often contain impurities such as metabolic byproducts and host proteins, which must be removed by efficient purification methods. This includes the use of advanced technologies such as continuous chromatography and ultrafiltration to separate the target bacteria from other impurities, thereby improving product purity and safety. Second, strict quality control is key to ensuring the safety and effectiveness of engineered bacterial therapies. Since bacteria may undergo genetic mutations or virulence recovery during production, regular genome sequencing and virulence testing are required to ensure that each batch of bacterial products is genetically stable and does not produce potential toxic side effects. The introduction of detectable virulence marker genes can monitor the virulence status of bacteria in the production process in real time, thereby identifying and excluding unqualified products at an early stage [225]. The sterility of the production process cannot be guaranteed, as live bacteria cannot be treated with traditional sterilization methods, which may lead to unintended bacterial contamination [14]. Precise molecular detection methods, such as gene sequencing, can be introduced to ensure the purity of the production process. Finally, transportation and preservation issues are also important challenges for engineered bacterial therapy. As living drugs, bacteria are required to remain active during storage and transportation. To combat this problem, optimized lyophilization techniques can preserve bacteria in a dehydrated state and reactivate them before use [226]. At the same time, adding protective agents such as trehalose or sucrose can improve the survival rate of bacteria under low temperature or dehydration to ensure their stability in long-term storage and transportation [227]. The development of new delivery vectors, such as nanoparticles, liposomes or magnetic materials, can not only enhance the targeted delivery effect of bacteria *in vivo*, but also provide protection for bacteria to avoid the loss of activity during transportation and complex environments *in vivo*. In summary, the enhancement of large-scale production and purification technologies, rigorous quality control processes, and optimized transport and preservation protocols are key steps in advancing engineered bacteria-based cancer immunotherapy to clinical applications. Through continuous improvement of these technologies, the potential of engineered bacteria in tumor therapy will be better realized in the future.

## 8 Future perspectives

Growing evidence supports the enrichment of bacteria within select tumors, creating a microecological network of interactions among tumor cells, immune cells, and microorganisms in the intratumoral environment [228,229]. These intratumor bacteria play a regulatory role in tumor development by forming microbiomes, with distinct bacterial taxa exhibiting opposite functions [230,231]. This underscores the importance of understanding the relationship between engineered bacteria and the host. Microbiomes with complex community structures are distributed in human skin, mouth, intestine, and tumors, engaging in intricate metabolic and immune interactions with their hosts. With a deeper understanding of the communication between bacteria and host, the design of immunotherapy with engineered live bacteria has become more expansive. In past studies, bacterial modification and immunotherapy strategies were relatively straightforward. To adapt to the complex communication pathways of both, much consideration should be given to how to leverage existing physiological processes. According to this principle, the entry points are mainly metabolic exchange, immune interaction, and bacterial colonization and migration.

In the regulation of metabolism, a modified EcN consistently transforms ammonia, a byproduct that builds up in tumors, into l-arginine. The presence of these bacteria in tumors led to an increase in l-arginine levels within the tumor, an elevation in the number of T cells infiltrating the tumor, and had significant combined effects with PD-L1 blocking antibodies in eliminating the tumor [232]. Therapeutic strains based on similar principles are already in clinical trials [233]. In immune interaction, the non-invasive administration method significantly improves treatment safety. The skin microbiome coexists peacefully with our tissues, without causing inflammation or triggering an infection. Some bacterial residents, such as the *Staphylococcus epidermidis* bacterium found on the skin, can elicit a highly targeted adaptive immune response. Chen et al. modified an *S. epidermidis* strain to produce melanoma tumor antigens and demonstrated its ability to stimulate anti-tumor immune reactions. This approach resulted in the generation of T cells that specifically targeted tumors, leading to reduced growth of both localized and metastatic melanoma without the need for bacterial injection. When combined with immune checkpoint inhibitors, these engineered skin bacteria prompted mice to reject established tumors [234]. For bacterial colonization and migration, the problem to be solved is to control the continuous colonization and efficient migration of bacteria from the gut to the tumor. It is difficult for engineered microbial organisms to colonize the hostile luminal environment. Russell et al. employed naturally occurring bacteria obtained from the fecal samples of conventionally raised mice, altering them to activate functional genes. Reintroducing these modified strains results in long-term establishment in the gut. Furthermore, engineered native *E. coli* leads to physiological changes and reversal of pathology over a period of months [235]. The synthesis of bacteria and the design of therapeutic modalities are intricately linked. *Lactobacillus reuteri* (Lr), transplanted orally into the mouse intestine, translocates into the tumor tissue and colonizes melanoma. Its metabolite I3A locally promotes interferon-γ-producing CD8^+^ T cells, enhancing the therapeutic effect of immune checkpoint inhibitors (ICI). Moreover, a tryptophan-rich diet can be metabolized by Lr to produce I3A, enhancing Lr- and ICI-induced antitumor immunity [236]. In the future, we hope that cancer immunotherapy using live bacteria will become a reality. With the support of synthetic biology and various therapeutic technologies, engineered bacteria are expected to link host life activities further to enable imaging, diagnosis and treatment of more diseases or physiological processes.

## Data availability

No data was used for the research described in the article.

## Declaration of competing interest

The authors declare that they have no conflicts of interest in this work.

## Appendix Supplementary materials

Image, application 1
