# Supplementary material for: Engineered bacteria: Strategies and applications in cancer immunotherapy
Source: Fundam Res. 2024 Nov 13;5(3):1327–45. doi: 10.1016/j.fmre.2024.11.001 (PMC12167902; doi:10.1016/j.fmre.2024.11.001)
Supplement: Supplementary file 1 [file mmc1.docx]

**Fig. 1. Basic strategies for targeting tumors by engineered bacteria**

(a) Hypoxia induces asd gene expression and bacterial normal growth. Aerobic induction of asd antisense mRNA production causes bacterial lysis. pPepT, hypoxia-induced promoter. pSodA, aerobic-induced promoter. asd, one of the critical genes in bacterial peptidoglycan synthesis. (b) Chemicals such as acid, purine and lactate induce bacterial enrichment in tumors. (c) Bacterial molecular interactions with tumor cells promote specific targeting. RGD, a tripeptide sequence consisting of L-arginine, glycine and L-aspartic acid. αvβ3, a type of integrin that is a receptor for RGD. (d) Use of magnetic fields to direct magnetotropic bacteria towards tumor enrichment. (e) Adjunctive methods such as macrophage transport and acoustic tweezers to control bacterial movement.

**Fig. 2. Multiple physical and chemical signals are input and trigger gene expression**

(a) Oxygen concentration in tumor microenvironment acts as a signal to regulate bacterial growth. FNR, umarate and nitrate reduction regulatory protein, transcription factor responsive to oxygen concentration. (b) Chemical molecules that trigger gene expression can often constitute an operon model. The diagram below illustrates the principle of the arabinose manipulator, in the absence of arabinose, gene expression is inhibited. The arabinose lifts the inhibitory effect of the repressor protein on gene transcription. IPTG, Isopropyl β-D-Thiogalactoside. (c) Light signals trigger gene expression. Near-infrared light shifts BphP1 from a non-activated to an activated state, promoting gene expression by inhibiting PpsR2 [1]. (d) Ultrasound triggers gene expression. The thermal effect of focused ultrasound inhibits the function of transcription factor TcI, which initiates gene transcription [2]. (e) Radiation triggers gene expression. Ion radiation-induced DNA breaks activate RecA through a series of reactions, which regulates gene expression by inhibiting LexA [3]. (f) Magnetic field triggers gene expression. The use of a magnetic field and nanoparticles to convert the magnetic field into heat signals regulates the expression of bacterial genes [4].

**Fig. 3. The basic units and structures that build genetic circuits**

(a) Feedforward is the pre-processing of the input signal. The green part is the correction of the AHL concentration, which is too high or too low to end up with no GFP fluorescence signal output. AHL, acyl-homoserine lactone. LuxR, an AHL-dependent transcriptional regulator. CI, lambda repressor. LacI, lac repressor [5]. (b) Feedback effect. The products of the loop eventually backfire directly or indirectly on the loop itself. Facilitation is positive feedback (pink), and inhibition is negative feedback (purple). (c) The toggle switch is designed by connecting simple circuits in series. This conjugate structure produces a mutual inhibitory effect thereby controlling the LacZ signal output [6]. (d) The recombinases recognize the *attB* (blue) and *attP* (orange) sites and flip the internal sequence to create permanent genetic memory. (e) AND gate. HrpR and HrpS are controlled by separate promoter inputs, and the *hrpL* promoter is activated only when both genes are expressed [7]. (f) OR gate. Inhibition of the transcriptional repression of RhaS or araC by arabinose or rhamnose initiates the expression of downstream genes [8].

**Fig. 4. Mechanisms of drug loading and release by engineered bacteria**

(a) Protein secretion system of Gram-negative bacteria. Signal peptides are critical for directing protein secretion. (b) The quorum sensing circuit realizes the periodic change of bacterial population, which leads to stable and sustained drug release. With the increase of the population, AHL induces the expression of lytic and drug proteins, causing bacteria to lyse and enter the next cycle. φX174 E, a bacteriophage lysis protein. HlyE, a pore-forming anti-tumor toxin [9]. (c) Multiple drug loading strategies of bacterial cell membranes.

**Fig. 5. Principles of cancer immunotherapy utilizing engineered bacteria**

(a) Immunogenicity of bacteria. Bacterial components activate Toll-like receptors that elicit the expression of anticancer cytokines through signal transduction. (b) The engineered bacteria express a variety of immunomodulatory factors. Synthetic gene circuits endow the engineered bacteria with the function of expressing cytokines and chemokines, causing immune cell activation and tumor cell apoptosis. (c) Immune checkpoint inhibitors. The quorum sensing circuit is used to lyse bacteria and stably release immune checkpoint inhibitors to regulate T cell activity [10]. (d) Cancer vaccines. After the phagocytosis and presentation of tumor antigens carried by the engineered bacteria by macrophages and dendritic cells, the effector T cells are activated to kill cancer cells, and the resulting memory T cells play a long-term anti-tumor function.

[1] N. T. Ong, E. J. Olson, J. J. Tabor. Engineering an e. Coli near-infrared light sensor, ACS Synthetic Biology, 7 (2018) 240-248. <https://doi.org/10.1021/acssynbio.7b00289>

[2] M. H. Abedi, M. S. Yao, D. R. Mittelstein*, et al.* Ultrasound-controllable engineered bacteria for cancer immunotherapy, Nature Communications, 13 (2022) 1585. <https://doi.org/10.1038/s41467-022-29065-2>

[3] S. Nuyts, L. Van Mellaert, J. Theys*, et al.* Radio-responsive reca promoter significantly increases tnfα production in recombinant clostridia after 2 gy irradiation, Gene Therapy, 8 (2001) 1197-1201. <https://doi.org/10.1038/sj.gt.3301499>

[4] X. Ma, X. Liang, Y. Li*, et al.* Modular-designed engineered bacteria for precision tumor immunotherapy via spatiotemporal manipulation by magnetic field, Nature Communications, 14 (2023) 1606. <https://doi.org/10.1038/s41467-023-37225-1>

[5] S. Basu, Y. Gerchman, C. H. Collins*, et al.* A synthetic multicellular system for programmed pattern formation, Nature, 434 (2005) 1130-1134. <https://doi.org/10.1038/nature03461>

[6] J. W. Kotula, S. J. Kerns, L. A. Shaket*, et al.* Programmable bacteria detect and record an environmental signal in the mammalian gut, Proc Natl Acad Sci U S A, 111 (2014) 4838-4843. <https://doi.org/10.1073/pnas.1321321111>

[7] L. Yang, A. A. K. Nielsen, J. Fernandez-Rodriguez*, et al.* Permanent genetic memory with >1-byte capacity, Nature Methods, 11 (2014) 1261-1266. <https://doi.org/10.1038/nmeth.3147>

[8] A. Wong, H. Wang, C. L. Poh*, et al.* Layering genetic circuits to build a single cell, bacterial half adder, BMC Biology, 13 (2015) 40. <https://doi.org/10.1186/s12915-015-0146-0>

[9] M. O. Din, T. Danino, A. Prindle*, et al.* Synchronized cycles of bacterial lysis for in vivo delivery, Nature, 536 (2016) 81-85. <https://doi.org/10.1038/nature18930>

[10] C. R. Gurbatri, I. Lia, R. Vincent*, et al.* Engineered probiotics for local tumor delivery of checkpoint blockade nanobodies, Sci Transl Med, 12 (2020) <https://doi.org/10.1126/scitranslmed.aax0876>
